# Magnetic Micellar Nanovehicles: Prospects of Multifunctional Hybrid Systems for Precision Theranostics

**DOI:** 10.3390/ijms231911793

**Published:** 2022-10-04

**Authors:** Margarida S. Miranda, Ana F. Almeida, Manuela E. Gomes, Márcia T. Rodrigues

**Affiliations:** 1 3B’s Research Group, I3Bs—Research Institute on Biomaterials, Biodegradables and Biomimetics, University of Minho, Headquarters of the European Institute of Excellence on Tissue Engineering and Regenerative Medicine, AvePark, Parque de Ciência e Tecnologia, Zona Industrial da Gandra, 4805-017 Barco, Guimarães, Portugal; 2 ICVS/3B’s—PT Government Associate Laboratory, 4710-057 Braga, Guimarães, Portugal

**Keywords:** hybrid nanosystems, magnetic polymeric micelles, polymeric micelles, magnetic nanoparticles, imaging, drug delivery, target delivery, nanotherapeutics, hyperthermia, magnetically assisted technologies

## Abstract

Hybrid nanoarchitectures such as magnetic polymeric micelles (MPMs) are among the most promising nanotechnology-enabled materials for biomedical applications combining the benefits of polymeric micelles and magnetic nanoparticles within a single bioinstructive system. MPMs are formed by the self-assembly of polymer amphiphiles above the critical micelle concentration, generating a colloidal structure with a hydrophobic core and a hydrophilic shell incorporating magnetic particles (MNPs) in one of the segments. MPMs have been investigated most prominently as contrast agents for magnetic resonance imaging (MRI), as heat generators in hyperthermia treatments, and as magnetic-susceptible nanocarriers for the delivery and release of therapeutic agents. The versatility of MPMs constitutes a powerful route to ultrasensitive, precise, and multifunctional diagnostic and therapeutic vehicles for the treatment of a wide range of pathologies. Although MPMs have been significantly explored for MRI and cancer therapy, MPMs are multipurpose functional units, widening their applicability into less expected fields of research such as bioengineering and regenerative medicine. Herein, we aim to review published reports of the last five years about MPMs concerning their structure and fabrication methods as well as their current and foreseen expectations for advanced biomedical applications.

## 1. Introduction

**Figure 1:**
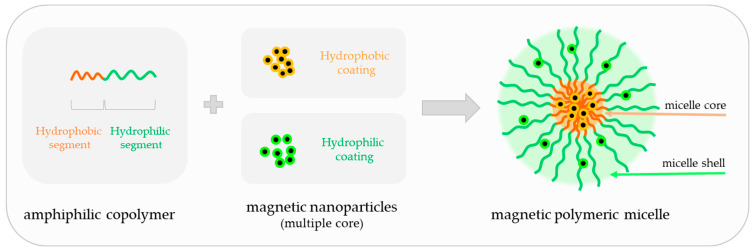
Schematic representation of a magnetic polymeric micelle (MPM). MPMs are formed by self-assembly of amphiphilic copolymers in the presence of multiple magnetic nanoparticle (MNPs) units. MNPs can have a hydrophilic (light green) or hydrophobic (light orange) coating to be incorporated into the hydrophilic (green) or hydrophobic (orange) segment of the polymeric micelle, respectively.

There is a worldwide growing demand for advanced medical nanotechnologies able to provide complementary functionalities mimicking complex biological processes while enabling biorecognition and bioresponsiveness to bring clinical benefits to patients. To meet these daunting challenges, hybrid nanoarchitectures such as magnetic polymer micelles (MPMs) offer unprecedented solutions at a cellular scale in fostering interactions with living entities and integrating extracellular environment dynamics. MPMs share de advantages of polymer micelles (PMs) and magnetic nanoparticles (MNPs) in a single nanoplatform with flexible capabilities (Figure 1).

Polymeric micelles (PMs) are nanosized colloidal particles built from amphiphilic copolymers that self-assemble in an aqueous solution above the critical micelle concentration (CMC). PMs are formed of a hydrophobic core and a hydrophilic outer shell and are characterized by a high loading efficiency of different cargos. As most conventional medicines share a hydrophobic nature, they can be accommodated in the hydrophobic core, whereas the hydrophilic shell may house complementary hydrophilic drugs and bioactive molecules in addition to providing colloidal stability and intrinsic stealth effect [1,2]. The shell is also responsible for environmental protection, limiting opsonin adsorption towards a longer circulation time or better blood stability and for active targeting through conjugation with ligands or antibodies against molecules and protein complexes at the cell membrane. This structural duality gives PMs high versatility and efficacy over other nanocarriers, packing multiple cargos with different properties into one system. Despite the multimodal potential, the impact of PMs in biomedicine can be greatly incremented by the inclusion of magnetic nanoparticles (MNPs) in the core or in the shell to produce magnetically responsive micellar structures enabling precision and noninvasive control over the spatiotemporal distribution of physical and chemical factors in cells, tissues, and living organisms.

In this review, magnetic micelles were defined as self-assembled core–shell nanostructures with possibilities for hydrophilic and/or hydrophobic loads in the polymeric segments and an MNP cargo within a single system, in contrast with amphiphilic polymer-coated single-core MNPs, which are sometimes referred to as magnetic micelles.

## 2. Fabrication of Magnetic Polymeric Micelles

The selection of amphiphilic copolymers is extremely important for micelle self-assembly and consequently for achieving innovative high-performance MPMs. The design of the core and shell may include synthetic and natural-origin polymers or molecules as building blocks that will support a dual architecture and render spatially independent moieties with complementary payloads (Table 1).

For a better understanding of the material properties and contribution to the hybrid system, amphiphilic polymers will be described accordingly to the hydrophilic/hydrophobic segment of the MPM they integrate.

### 2.1. Types of Polymers Used in the Shell of Magnetic Polymeric Micelles

#### 2.1.1. Synthetic Polymers

Synthetic polymers have been widely studied in bioengineering due to their well-defined chemical structure, batch-to-batch uniformity, and biocompatibility [3,4]. They are also recognized for their high purity, suitable mechanical properties, and nontoxicity.

The most investigated polymer for the MPM shell is poly(ethylene glycol) (PEG). PEG is a highly versatile polymer with excellent solubility in aqueous media and chemical labiality for chemical modification with different functional groups [5]. PEG provides both the stealth effect and immune protection by reducing the adhesion of opsonins present in the blood as well as MPM uptake by phagocytic cells [6]. A potential alternative to PEG is polysarcosine (PSar), a nonionic polypeptoid based on the amino acid sarcosine, i.e., *N*-methylated glycine, found in muscles and other tissues [7]. PSar presents PEG-like properties including high water solubility and protein resistance, low cellular toxicity, and a nonimmunogenic character. A less conventional block is triethylene glycol monomethyl ether (TEGME). TEGME is a hydroxypolyether that has been used to bind to the poly(phenyl isocyanide (PPI) block to afford a water-soluble amphiphilic copolymer [8].

Smart polymers can undergo physical or chemical modifications in response to a change in environment or a precise stimulus such as pH or temperature (revised from [9]). Poly amino acids and esters (e.g., poly(glutamic acid) (PGA), poly(2-azidoethyl-L-glutamate) (PAELG), poly(β-amino ester) (PAE) as well as polyolefines (e.g. poly(acrylic acid) (PAA)) have been investigated to increase the hydrophilicity of the shell to control MPM responsiveness to pH variation. For gene delivery, the micelle corona is typically constituted by poly(ethylenimine) (PEI). The amino groups available in PEI are responsible for their highly positive charge density and the binding to negatively charged molecules comprising short DNA or RNA polymers. PEI has been considered one of the most efficient nonviral gene delivery vectors [10,11] despite its reported nonbiodegradability and cellular toxicity. To circumvent PEI limitations, cationic spermine (Spm) and poly(aspartic acid)-dimethylethanediamine (PAsp(DMA)) were proposed for delivering nucleotides [12]. Spm is an aliphatic polyamine bearing multiple amino groups holding important roles in the metabolism of eukaryotic cells [13]. Considering its endogenous function, it is expected that Spm will render improved biocompatibility over that of other synthetic molecules. Polymers sensitive to temperature, for instance poly(N-isopropylacrylamide) (PNIPAM), have been used for thermal-controlled drug release. PNIPAM presents a reversible hydration/dehydration state across the lower critical solution temperature (LCST, 32 °C), whose behavior is associated with conformational changes of the polymer chains induced by temperature variation [14,15]. Other polymers, for instance poly(2-hydroxymethyl) methacrylate (PHEMA), have been combined with platinum to generate a polymeric prodrug that can be further assembled into a micellar nanocomplex for enhanced accumulation and efficacy of tumor-oriented drugs [16].

#### 2.1.2. Natural-Origin Polymers

Natural-based polymers are biomaterials obtained from sustainable and renewable sources, namely plants, animals, or fungi. Most natural-origin polymers are cost-effective, easily available, biodegradable, and nontoxic with vast applications in pharmaceutics and biotechnology. Natural polymers have been widely investigated in different nanoarchitectures (revised from [17]). Among these, polysaccharides such as hyaluronic acid (HA) [18,19,20], chitosan (CHI) [21,22], and dextran (DEX) [23] and proteins such as lactoferrin (LF) [24] have been proposed for the development of MPM due to the presence of specific moieties that promote cell recognition and interactions with cells [25].

HA is naturally found in tissue matrices having a crucial role in tissue structure, cell motility and adhesion, and proliferation processes. HA is already applied in clinics for diagnosis, in ophthalmological and ontological surgeries, in the treatment of arthritic patients, and also in the cosmetic regeneration and reconstruction of soft tissue [26,27]. Furthermore, HA is chemically versatile and can be modified in a myriad of forms to ameliorate its physico-chemical properties and biological functionality.

CHI is a polysaccharide produced by the deacetylation of chitin obtainable from marine biological structures. CHI is one of the few cationic polymers available for electrostatically binding with anionic bioactive compounds. CHI contains amino and hydroxyl groups on its backbone, permitting its conjugation with bioactive molecules under physiological conditions. Like HA, CHI can be easily modified to improve the targeting efficiency, respond to environmental stimuli, and obtain value-added delivery systems by increasing the solubility and embedding efficiency of hydrophobic therapeutic agents. Based on such findings, amphipathic CHI derivatives have been developed by modifying CHI with hydrophobic octyl, hydrophilic quaternary ammonium, and PEG groups to fabricate a drug delivery carrier with enhanced solubility and controlled payload release [22].

MPM shells have also been fabricated with DEX, which can be obtained from the lactic acid bacteria *Leuconostoc mesenteroides* and commercially produced from sucrose [28]. Because DEX possesses abundant hydroxyl groups, it is a water-soluble polymer with chemical modification potential for the transportation of drugs, proteins, and other bioactive agents. Hydrophobic chains, stimuli-sensitive chains/groups, and drugs have been conjugated to DEX to obtain self-assembling derivatives. DEX is resistant to protein adsorption and is clinically applied as a plasma expander and as an antithrombotic agent to reduce blood viscosity [28,29].

Along with polysaccharides, proteins have served as micellar shells. LF is a non-heme iron-binding glycoprotein derived from milk and other mammalian fluids with antimicrobial activity [30]. LF has an unusually high isoelectric point (pI > 8) and therefore tends to be cationic at neutral pH. LF has been described as minimizing the possible interaction of MPMs with serum proteins, extending their systemic circulation [24].

### 2.2. Types of Copolymers Used in the Core of Magnetic Polymeric Micelles

#### 2.2.1. Synthetic Polymers

Poly(ε-caprolactone) (PCL) is a commonly used polymer for the MPM core, together with polylactic acid (PLA) and poly(lactide-*co*-glycolic acid) (PLGA). These polyester-based polymers are approved by the United States Food and Drug Administration (FDA) for several biomedical applications and share the characteristics of hydrophilic synthetic polymers (Section 2.1.1), comprising well-defined chemical structure, biocompatibility, and nontoxicity. Since most molecules with therapeutic value have hydrophobic behavior, other hydrophobic blocks like poly(L-aspartic acid) (PAsp) derivatives, poly(4-vinylpyridine) (P4VP), and poly(styrene) (PS) have been proposed to transport MNPs and drugs inside MPMs. In particular, poly(2-methoxy-2-oxo-1,3,2-dioxaphospholane) (PHEP) and poly(acrylamide-*co*-acrylonitrile) (P(AAm-*co*-AN)) transit from a hydrophobic state to a water-soluble disassembled state upon heating, enabling temperature-responsive payload release which is of special interest in drug delivery during hyperthermia treatment [31,32].

#### 2.2.2. Small Molecules

Palmitic acid (PA), 1,2-distearoyl-*sn*-glycero-3-phosphoethanolamine (DSPE), cholic acid (CA), and aminoethyl 5β-cholanomide (CAM) are small hydrophobic molecules utilized as lipid moieties in the assembly of MPMs. The balance of phospholipids in cells is maintained by PA, the most common saturated fatty acid found in the cell membrane of animals, plants, and microorganisms. Likewise, phosphatidylethanolamines are available in cell membranes and may contribute to improved magnetic micelle-cell communication. With different biological roles, CA and 5β-cholanic acid are bile acids responsible for facilitating the digestion of dietary fats serving as micelle-forming surfactants. CA is FDA approved for the treatment of bile acid synthesis disorders. CAM is derived from 5β-cholanic acid that has been chemically changed with a terminal amino group to be conjugated with polymers containing carboxylic acids such as HA [33].

#### 2.2.3. Proteins

Despite the potential for MPM assemblies, protein reports are scarce. The only exception is Zein, a prolamine that occurs specifically in cereals [34]. Zein is the major storage protein in corn and has been considered a waste protein until recently. Its unique solubility in aqueous alcohol solutions and deficit in basic and acidic amino acids, especially tryptophan and lysine, have been recently appreciated for drugs and bioactive molecules delivery and tissue engineering [35,36,37].

### 2.3. Intrinsically Amphiphilic Natural Polysaccharides

Up to the present time, levan was the only intrinsically amphiphilic natural polysaccharide described with obvious potential for MPM assembly [38]. Levan is a fructose homopolymer derived from microorganisms and some plants with anti-tumor and anti-infection activities [39]. Levan presents a hydrophobic moiety and the methylene group in the furanoside moieties, and its amphiphilic character has shown prospects for water-forming nanoparticles and the delivery of proteins and peptides [39,40].

### 2.4. Magnetic Nanoparticles

#### 2.4.1. The Impact of Magnetic Compliance 

MNPs are a class of nanomaterials characterized by their magnetic responsiveness to external magnetic fields. MPMs typically incorporate iron oxide-based MNPs, namely, superparamagnetic iron oxide nanoparticles (SPIONs) such as magnetite (Fe_3_O_4_) [8]. In contrast with other MNPs, the superparamagnetic behavior allows SPIONs to lose magnetism and re-disperse when the magnetic field is removed, which is an appealing feature for the manipulation and navigation to specific sites under an external magnetic field.

The basic principle of magnetic field-based manipulation relies on magnetic field strength, a magnetic field gradient, and a susceptible difference between the magnetically responsive material and the cells or tissues [41]. The magnetic field applied can be constant, also referred to as static or stationary magnetic field and provided by permanent magnets, or alternating, induced by charge movements in solenoids or electromagnets. Unlike static magnetic fields, alternating magnetic fields (AMFs) vary in time and can be divided into low or high frequency depending on how slow (low) or fast (high) the magnetic field varies in time. Depending on the imaging devices, the magnetic fields in magnetic resonance imaging (MRI) go up to 7 T for several minutes while musculoskeletal treatment modalities with magnetotherapy equipment (e.g., Globus XL) vary between 1.5 mT to 10 mT applied for up to 3 h per session.

Magnetic fields demonstrate excellent tissue penetration and have a low interference with the biological environment due to the not inherently magnetic nature of cells and tissues, which are powerful arguments for taking advantage of magnetic field-based technologies in the development of multifunctional hybrid systems. The contactless actuation of the magnetic field minimizes possible harmful effects that could reduce cell integrity and viability. Moreover, the magnetic field is poorly influenced by internal and external features such as ionic strength, surface charges, pH, and temperature, which can be advantageous for human-driven applications. Finally, magnetic fields can be generated from simple use and inexpensive instrumentation (e.g., permanent magnets) that can support and facilitate the exploitation and commercialization pathways of magnetic targeting into clinical modalities.

The strong magnetic moment of MNPs has been explored for visualization, tracking, and monitoring in imaging techniques such as MRI and magnetic particle imaging for assisting with diagnosis modalities. This unique feature of MNP-based probes could transform disease detection and imaging-guided therapies into precision delivery and targeted theranostic platforms with promising interventions in the management and treatment of pathological conditions.

#### 2.4.2. Magnetic Nanoparticles Synthesis

MNPs can be synthesized by wet chemistry, microfluidic reactors, or biogenic synthesis (revised from [42,43,44]) to produce nanoparticles with well-controlled size, distribution, and shape. The synthesis route also determines the hydrophilic/hydrophobic nature of the MNP coating, and thus the MNP integration within one of the MPM segments.

In MPM assemblies, thermal decomposition and co-precipitation are the most commonly used methods of producing MNPs. Thermal decomposition produces hydrophobic MNP with tunable sizes (5–22 nm), narrow size distribution, and high crystallinity, holding scalability potential [45,46]. On the other hand, co-precipitation, which is a simple and rapid procedure, results in the synthesis of MNPs with high polydispersity and poor crystallinity [2,47,48]. Typically, MNPs are synthesized first and then co-assembled with amphiphilic copolymers into MPMs. Nevertheless, a study by Bastakoti et al. reported the in situ synthesis of SPIONs by co-precipitation after micelle assembly with poly(styrene-*block*-acrylic acid-*block*-ethylene oxide) (PS-*b*-PAA-*b*-PEG) [2]. The chelating behavior of the carboxylic acid groups from the PAA segment provides the reaction sites for iron ions and controls the crystal overgrowth during the co-precipitation reaction. This unconventional approach resulted in an increase in the hydrodynamic diameter of the MPMs in comparison with nonmagnetic micelles and a higher polydispersity index (PDI of 0.3) due to the formation of secondary nano and micro aggregates [2]. Even though the in-situ synthesis of MPMs has been scarcely reported, the control of the properties such as dimension, shape, and magnetic responsiveness are likely more pertinent to improving MPM functionalities and effectiveness as cutting-edge nanovehicles.

#### 2.4.3. Shape and the Core Composition of MNPs

The shape and the core composition of MNPs have been explored in MPM production anticipating efficiency improvements for diagnostic and therapeutic actions. Although the MNPs used in the MPM fabrication are often spherical, other shapes have been prepared by playing with synthesis parameters [49,50]. Cube-shaped SPIONs evidenced higher heating efficiency and enhanced MRI contrast in comparison with sphered SPIONs due to their value-added magnetic properties [51,52,53]. Accordingly, cubic MnFe_2_O_4_-based MPMs exhibited a higher negative contrast enhancement of MRI signals (*r*_2_ = 373 mM^−1^ s^−1^) in comparison with spherical MnFe_2_O_4_-based MPMs (*r*_2_ = 321 mM^−1^ s^−1^) highlighting the morphology and shape of SPIONs as critical aspects for the development of diagnostic imaging agents [49]. MPMs have also been produced using SPIONs doped with Zn and/or Mn known as ferrite nanoparticles, where iron is partially substituted by other metallic elements (e.g., *M*Fe_2_O_4_, *M* = Zn; Mn) [49,54,55]. These elements have been shown to increase the magneto-thermal capability and the performance of MRI contrast agents relative to commercially available contrast agents based on pure SPIONs (Fe_3_O_4_) [56]. MnFe_2_O_4_-, Zn_1.15_Fe_1.85_O_4-_, and Mn_0.6_Zn_0.4_Fe_2_O_4_-based micelles have been constructed for imaging purposes [49,54,55]. Interestingly, Zn_1.15_Fe_1.85_O_4_ micelles have also shown value for magnetic delivery and Mn_0.6_Zn_0.4_Fe_2_O_4_ micelles for hyperthermia treatments, supporting the multifunctional identity of magnetic-based nanosystems. Nonetheless, comparative studies are still missing for hierarchically classifying the important features influencing contrast imaging properties, heating capability, and half-life in circulation.

#### 2.4.4. Biocompatibility of MNPs 

The low tendency of SPIONs to form particle aggregates after magnetic field removal, rendered by their superparamagnetic nature, is highly relevant to biomedicine approaches and contributes to a significant decrease in toxicity in living environments [57]. Similar to other metallic nanoparticles, the toxicity of SPIONs is dependent on various factors including size, surface chemistry, concentration, the method of administration, and biodegradability [58,59,60,61,62,63,64], and may be related to oxidative stress and iron-mediated radical formation [65]. Nonetheless, the potential toxicity effects of SPIONs below 100 µg mL^−1^ have been considered neglectable on several cell lines [58,60].

Although the interactions between MNPs and cells or tissues are not comprehensively elucidated, and nor are the long-term biological impacts in humans, studies have demonstrated that MNPs are engulfed in cell endosomes that later fuse with lysosomes, where an acid-induced degradation occurs [66]. The iron ions released from the SPION degradation join the intracellular iron pool and the innate iron metabolic pathway. Ultimately, these ions are recycled and used for hemoglobin synthesis after uptake by erythroid precursor cells [67].

In response to foreign nanoparticles, cells can also orchestrate autophagy, with crucial signaling in processes like infection, inflammation, polarization, and tumor cytotoxicity, to assist in important functions including antigen presentation, lymphocyte homeostasis, and the secretion of immune factors. Disputable outcomes of MNP-induced autophagy suggest that the autophagic response of macrophages may correlate with cytokine cascades and inflammatory response, while others indicate a protective autophagy response of SPIONs in monocytes and macrophages [68,69]. 

Immune cells are sensitive to the presence of MNPs and respond by either releasing inflammatory mediators or stimulating anti-inflammatory functions. Immune activation can be useful, for instance for enhancing antitumor immunity by reversing the M2-like phenotype of tumor-associated macrophages, which serve roles in inhibiting inflammation and promoting tumor development. Macrophages exposed to ferumoxytol (Feraheme^®^) displayed increased mRNA associated with pro-inflammatory responses, suggesting that ferumoxytol could be applied “off label” to potentiate macrophage-modulating cancer immunotherapies [70]. Additionally, iron oxide@chlorophyll clustered nanoparticles modified with 4-carboxyphenylboronic acid were reported to reprogram tumor microenvironment in combination with photo and chemodynamic therapies by the inactivation of programmed death-ligand 1 and suppression of M2-like macrophages accumulation [71].

On the other hand, the interaction of human macrophages with dextran-coated [72,73] and silica-coated [72] SPIONs did not trigger the release of pro-inflammatory molecules as IL-12, IL-6, TNF-α, and IL1-β, suggesting that the immunomodulatory capacities of MNPs can be also utilized to potentiate new imaging scenarios, regulate the phenotype transition of immune cells, or to create an environment conducive for tissue regeneration preventing persistent inflammatory triggers. In a work by Wu et al., a fibroblast growth factor (bFGF)-loaded Fe_3_O_4_ nanoparticle accelerated wound healing through M2 macrophage polarization and increased cell proliferation in a full-thickness wound murine model [74].

Thus, controlling the design of MPMs, in particular, the surface functionalities of SPIONs to meet a particular application could tackle the biosafety concerns. Furthermore, the small dimension of MPMs enables optimal in vivo delivery, avoids rapid renal clearance, and improves MPM’s safety and effectiveness [75,76]. These features together with a high loading capacity and magnetic navigation foresee MPMs as compelling smart structures for state-of-the-art medical diagnosis and therapeutic purposes [47,48], capitalizing on MPM’s translational success.

### 2.5. Self-Assembly Architectures of Amphiphilic Copolymers

Amphiphilic copolymers were designed to self-assemble into MPMs with di-block, tri-block, multi-block, star-like, and graft copolymer architectures (Table 2).

The hydrophobic moieties can be introduced by grafting them as side groups along the polymer chains or via coupling to end groups (end-to-end coupling strategies), using chemical tools (e.g., amidation, esterification, click chemistry) or polymer synthesis (e.g., ring-opening or living radical polymerization).

The nanocarriers based on traditional di-block copolymers are characterized by poor structural tunability. The combination of different block types into mixed micelles enables the design of nanostructured materials with increased architectural complexity, improved control over micellization, and enhanced functionality. An example is the use of a star-like block copolymer with PLGA and PEG arms for the fabrication of MPM aiming at quercetin (QCT) drug delivery [77]. Star polymers contain more end-groups than linear polymers, thus presenting higher hydrophilicity, and when used as drug nanocarriers, they also show higher drug loading efficiency [78]. Moreover, the demand for multifunctionally competent MPMs has led to the arrangement of linear polymers with different generations of dendrimers referred to as telodendrimers (Table 2). The MPM assemblies with telodendrimers found applicability in MRI and fluorescent dual imaging joining telodendrimer dendritic oligo-cholic acid-*block*-poly(ethyleneglycol) ((CA)_4_-Lys_3_-PEG) with SPIONs and Nile Red, a model hydrophobic dye. (CA)_4_-Lys_3_-PEG was designed by linking the end of PEG with the second generation of dendritic polylysine (PEG-Lys_3_) to which CA molecules were coupled [79].

The self-assembly of copolymers and the integration of MNPs into MPM involves the optimization of the hydrophilic/hydrophobic balance of the block copolymer, MNP surface ligands, and the type of organic solvent used to dissolve copolymers and MNPs [80]. The colloidal stability of MPM is of critical importance for preventing the premature release of drugs and prolonging the blood circulation time. Cross-linking methods have been used to fix the core or the shell of MPM with organic molecules. Although covalent cross-linking can be employed in both the hydrophobic and hydrophilic segments, cross-linking of the shell can lead to inter-micellar cross-linking, loss of shell fluidity, and polar affinity causing a decreased stealth effect.

Traditional organic cross-linking strategies involve toxic and expensive organic molecules as well as complicated and time-consuming procedures. Alternatively, organosilica cross-linking is performed under mild conditions and involves easier processing. This strategy was performed by Yang et al. to lock the PCL core using a 3-mercaptopropyltrimethoxysilane cross-linking agent under alkaline conditions [81]. The resultant MPM exhibited excellent stability in biological fluids. 

Using a double cross-linking approach, Bauer et al. [82] developed MPM from SPIONs and PCys(SO_2_Et)-*b*-PSar copolymer. These MPMs were cross-linked with dihydrolipoic acid enabling both disulfide bond formation in the core and direct grafting onto the SPIONs surface through the carboxylic group of dihydrolipoic acid. This approach was successful to improve the stability of the SPIONs in the MPM.

**Figure 2:**
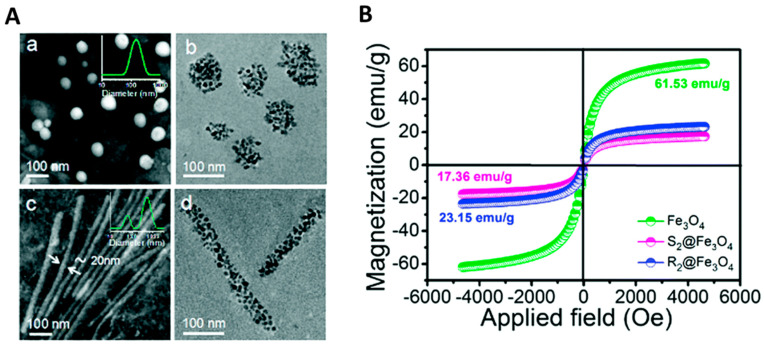
The influence of the shape in the magnetization of magnetic polymeric micelles. (**A**) TEM images of micelles produced with PBA-PEG-PCL@Fe_3_O_4_ with spherical (**a**,**b**) or rod-like (**c**,**d**) morphology. Images in (**a**,**c**) represent micelles without Fe_3_O_4_ while (**b**,**d**) refer to magnetic polymeric micelles. In panels (**a**,**c**), the inserted images indicate the nanoparticle size distribution. (**B**) The magnetization loop was performed on the magnetic particles (Fe_3_O_4_) and the spherical (S_2_@Fe_3_O_4_,) and rod-like (R_2_@Fe_3_O_4_) micelles at room temperature by vibrating sample magnetometry. Adapted with permission from Ref. [85]. Copyright 2022, The Royal Society of Chemistry.

MPMs generally exhibit a spherical morphology ranging from 20 to 500 nm [21,49,79,83,84] but, rod bacterium-like MPMs have been developed (Figure 2) [85] with a diameter of 20 nm and length of 600 nm combining PCL-PEG, SPIONs, and doxorubicin (DOX). Interestingly, rod MPMs were shown to present a higher loading content of DOX together with a higher saturation magnetization (23.15 emu g^−1^) than spherical MPM (17.36 emu g^−1^). Moreover, rod-shaped MPMs have an extensive blood circulation time (>24 h) and are more easily internalized by HepG2 and A549 cells than their spherical counterparts [85].

Micelle size is influenced both by the size [21,49,83,84] and the clustering [86] of loaded SPIONs. For a given SPION-loaded amount, increasing SPIONs size leads to an enlargement of MPMs. The amount of loaded SPIONs also influences the micelle diameter, as elegantly investigated by Jiang et al. [86]. In this work, the SPION-free micelles presented a diameter of 24 ± 3 nm, and the diameters increased to 80 ± 13, 100 ± 9 and 108 ± 8 nm, respectively, when SPIONs were loaded into the micelles by weight ratio of 15, 30, and 50% [86].

### 2.6. Techniques for the Preparation of Magnetic Polymeric Micelles

**Figure 3:**
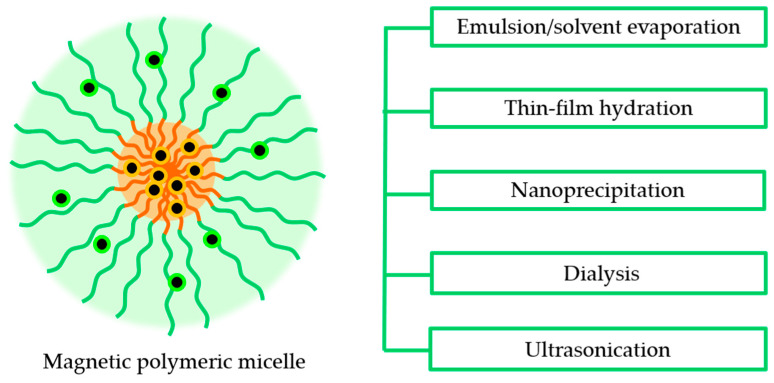
Methods for the production of magnetic polymeric micelles.

The shape-controlled and highly stable MPMs with narrow size distribution were successfully achieved through five distinct routes: (i) emulsion–solvent evaporation, (ii) thin film rehydration, (iii) nanoprecipitation, (iv) dialysis, and (v) ultrasonication (Figure 3). The selection of one of these routes depends on the solubility of the copolymer considering that the MNPs are commonly hydrophobic and thus are dissolved in an organic solvent. Likewise, the hydrophobic/hydrophilic nature of drugs to be incorporated in the core or shell of the MPM, respectively, dictates that hydrophobic drugs be included in the organic phase while the hydrophilic drugs are dissolved in the aqueous solution.

In the emulsion–solvent evaporation method, the co-polymer and MNPs are dissolved in an organic solvent. The resulting mixture is added to an aqueous solution under stirring or sonication to which surfactants may be added to increase the stability of the emulsion. The organic solvent is then evaporated to form the MPMs. In a study by Karami et al. [76], naproxen (NPX)-loaded MPMs were successfully prepared using this method. The organic phase containing the dissolved PCL-*b*-PEG copolymer, naproxen, and SPIONs was injected into the aqueous phase (polyvinyl acetate 0.5% *w*/*v*) under homogenization to obtain an emulsion followed by the chloroform evaporation.

In the case of copolymers with phospholipids in the hydrophobic block, thin-film hydration was used. Polymeric micelles of DSPE-*b*-PEG have been produced with MNPs and paclitaxel (PTX) loads by dissolving the copolymer and MNPs in a volatile organic solvent that was then evaporated by rotary evaporation to produce a thin film [87,88]. The film is rehydrated and vortexed vigorously to produce MPMs.

The nanoprecipitation and dialysis methods have a similar procedure. The copolymer and MNPs are first dissolved in a water-miscible organic solvent and added to the aqueous phase under stirring. With the nanoprecipitation method, the MPMs are readily formed, and the organic solvent is removed by evaporation or dialysis. In the dialysis method, the mixture is placed into a dialysis bag and immersed in water for several hours, inducing the MPM assembly through solvent exchange [83]. Unlike the nanoprecipitation method in which the MPMs are produced in a simple and fast manner, the dialysis method can be time-consuming and requires a great amount of water, resulting in the formation of a large volume of waste liquid.

Ultrasonication is normally used for water-soluble copolymers. MNPs are added to the copolymer, and an ultrasonication procedure allows the formation of MPM after removing the organic solvent. In a study by Park et al. [38], SPIONs in hexane were added to levan, and the mixture was moved to an ultrasonic water bath. The organic solvent was then removed by heating.

## 3. Magnetic Micelles in Cell and Tissue Targeting 

The interactions established between MPMs and living entities define the accomplishment or failure of MPMs’ applicability in clinics. MPMs exhibit unique and finely tunable properties with promise for MRI, magnetic therapeutic delivery and responsive release, combined thermal-chemotherapy, and nucleotide delivery, ultimately assisting with the integration of diagnosis and therapy in a single entity (Table 3).

Circulating MPMs for imaging purposes need minimal cell or biological fluid interplay before reaching the site of interest. However, the strategies for local administration towards tissue targeting and immunotherapies may consider other requirements for micelle–cell membrane interfaces. More than a passive nanocarrier, the MPM can be viewed as a therapeutic agent itself. MPMs can be engineered to engage with cell surfaces, assisting with micelle internalization, or to extracellularly influence intracellular pathways. In both cases, MPMs may support cell-free therapies through direct cell guidance.

The outer shell of MPM can be modified to enable cellular interactions with the conjugation of specific moieties like peptides or proteins (e.g., antibodies, receptors), small molecules (e.g., carbohydrates, folate), low-density lipoproteins, or aptamers. The surface changes are designed for boosting the selectivity to specific cells and for site-specific drug release; increasing intracellular drug concentrations, reducing drug toxicity and adverse side effects compared with untargeted MPMs and isolated commercial drugs.

To facilitate the homing of nanotherapeutics to the target tissue and aiming for desirable functionality, ligands to cell receptors are preferential choices for targeting moieties at MPMs. These molecules participate in receptor-mediated endocytosis [24] or activation/inactivation of protein complexes formed by molecular recognition with influence over intracellular signaling [54]. The most promising ligand-receptor moieties are specific to the cell of interest or are differentially expressed in healthy versus pathological stages.

Folic acid (FA) is a model targeting ligand for anticancer strategies as a result of the overexpression of folate receptors in cancer. To improve the specificity of MPMs, a multi-block polyurethane was designed with an L-cysteine-derived chain extender to allow a FA post-conjugation via click chemistry [89]. Under acid environments like the ones found in tumor niches, the clicked FA ligands can be switched on for activated tumor targeting and cellular uptake, enabling the release of MPMs within tumor cells to approach precision anti-cancer therapies. Other molecules such as galactosyl and lactosyl sugar units were described to interact with asialoglycoprotein receptors, which are overexpressed on hepatocellular carcinoma cells (HepG2), due to their lectin-recognition properties. The galactosyl and lactosyl were conjugated to a polypeptide block of PCL-*b*-PAELG further loaded with an anticancer drug, DOX and SPIONs and efficiently transported into HepG2 cells [83].

**Table 3:** Magnetic micelles produced for different applications.

Amphiphilic Co-Polymer	MNPs	Therapeutic Agent	Target Ligand	Approach	Outcomes	Ref.
Imaging						
GCPQ	Fe_3_O_4_	-	-	MRI (liver vasculature)	- High *T*_2_ contrast with a spatial resolution for detailed liver vasculature - Blood half-life of 28.3 min - Preferential accumulation in liver and spleen	[21]
DSPE-PEG	Fe_3_O_4_	-	-	MRI (liver)	- High *T*_2_ contrast up to 3 h in the liver - Low toxicity	[87]
PDLA-*b*-PEG/PLLA-*b*-PEG	MnFe_2_O_4_ (s,c) MnFe_2_O_4_@Fe_3_O_4_	-	-	MRI	- Stereocomplexation micelles with improved stability - Cubic SPION-loaded micelles with higher *T*_2_ contrast imaging than the ones with spherical- or core-shell.	[49]
P4VP-*b*-PEG	Fe_3_O_4_	-	-	MRI	- Fabrication of worm-like micelles with a larger number of loaded SPIONs - Higher *r*_2_ in worm-like micelles than in free SPIONs - Shape favors MPM circulation in blood	[90]
(CA)_4_-Lys_3_-PEG	Fe_3_O_4_	-	-	MRI	- Ultra-small size micelles (<40 nm) - MRI sensitivity superior to that of free SPIONs	[79]
WPU-BPLP-WPU	Fe_3_O_4_	DOX	Y_1_R	MRI	- Darker *T*_2_-weighted signal with MPMs - Improved blood circulation of iron in MPM (half-live of 69.3 h) than in SPIONs (5.4 h)	[91]
(CA)_2_-Lys-(PAsp(DMA))	Fe_3_O_4_	-	-	stem cell labeling MRI of transplanted NSCs	- In vivo MRI tracking of NSCs without detrimental effects - Cationic micelles (+15 mV) are safer and more efficient for cell labeling than neutral micelles	[75]
Levan	Fe_3_O_4_	-	-	MRI cell tracking intracellular magnetic actuators	- Use of a natural polysaccharide block for improved biocompatibility - MRI sensitivity superior to that of free SPIONs - Dual mode imaging probes combining SPIONs with quantum dots or gold nanoparticles	[38]
Theranostic potential						
Therapeutics delivery and responsive release						
PLGA-PEG	Fe_3_O_4_	QCT	-	drug delivery	- Star-like block polymer - Drug loading of 13.4% and loading efficiency of 68% - Drug release of 37% after 7 h, and 90% after 72 h	[77]
PCL-*b*-PEG	Fe_3_O_4_	NPX	-	magnetic drug delivery	- Smaller micelles (<150 nm) are more efficient for brain-targeting with a higher accumulation of NPX than larger micelles (~240 nm) or free drug - Magnetic field of 0.4 T externally applied to direct micelles to the brain - Prolonged blood circulation in comparison to free drug	[76]
Zein-LF	Fe_3_O_4_	DAS	-	magnetic drug delivery pH-release	- Faster release of DAS at pH 5 > pH 7.4 - Increased cytotoxicity against MDA-MB-231 cells - A magnetic field of 0.5 T significantly reduced the IC_50_ of DAS-loaded MPM (22.41 µM) to 16.48 µM (IC_50_ free drug = 30.43 µM) - in vitro serum stability and hematocompatibility	[24]
PCCL-*b*-PEG	Fe_3_O_4_	PTX	-	magnetic drug delivery magnetic and pH-release	- High PTX loading - Faster release of PTX at pH 6.5 > pH 7.4 - Low in vitro and in vivo cytotoxicity - Higher inhibition of the tumor rate than with a magnetic field of 1.7 T in PTX-loaded MPM (45.23%) than with non-magnetic micelles (30.78%) or free-PTX (7.12%) - Effective tumor-specific cell targeting with magnetic field	[92,93,94]
PCL-*b*-PEG	Fe_3_O_4_	QCT	-	pH-release	- Faster release of QCT at pH 5.3 > pH 7.4 - Drug loading of 17.1% and encapsulation efficiency of 95.9% - Low toxicity (mitochondrial assays)	[95]
Octyl-g-HTCC/Octyl-g-PEG-HTCC	Fe_3_O_4_	PTX	-	pH-release	- Faster release of PTX at pH 5.0 > pH 6.5 > pH 7.4 - Drug loading of 19.71% and encapsulation efficiency of 88.71%	[22]
P(NIPAAm-*co*-DMAAm-*co*-UA)	Fe_3_O_4_	Hesp	-	pH-release	- Faster release of QCT at pH 6.6 > pH 7.4	[96]
PSar-*b*-PCys(SO_2_Et)	Fe_2_O_3_	Iron (Fe_2_O_3_)	-	redox-release	- High iron loading (33 wt%) - MPM degradation mediated by GSH (10µM–100mM) - Induce macrophage activation in vitro and in vivo	[82]
PCL-*b*-PEG	Fe_3_O_4_	DOX	-	redox-release	- Use of a simulation method to calculate loading (10%), and encapsulation efficiency (60%) - DOX release increased from 40% to 60% with dithiothreitol (10mM)	[97]
PLA-PEG/PLA-CHI-Spm	Fe_3_O_4_	siRNA PTX	FA T7 peptide	dual therapeutics delivery pH-release	- Higher release at pH 6 > pH 7.4 - Encapsulation efficiency: 68.52% (siRNA) and 38.11% (PTX) - Lower IC_50_ (35.4 nM) in MPM combining FA + T7 peptide targeting	[98]
Imaging/Therapeutics delivery/Combined therapies						
PCL-*b*-PEG	Fe_3_O_4_	TAM	-	MRI drug delivery	- Drug loading of 8.14% and encapsulation efficiency of 52.19%	[99]
PS-*b*-PAA-*b*-PEG	Fe_3_O_4_	DC	-	MRI/optical imaging drug delivery	- MNPs synthesized by co-precipitation on the PAA shell - Incorporation of a positively charged drug on the PAA shell	[2]
DSPE-PEG	Fe_3_O_4_	PTX	-	MRI drug delivery	- SPIONs do not influence cell viability up to 0.8 mg mL^−1^ - Good *T*_2_-weighted image contrast - Significant increase of apoptotic activity in tumor mouse models	[88]
OCL-Bz-*b*-PEG	Fe_3_O_4_	QCT	-	MRI magnetic drug delivery	- Drug loading of 3.5% and encapsulation efficiency of 70% - *r*_2_ values of 137 mM^−1^ s^−1^ (SPIONs) and 246 mM^−1^ s^−1^ (MPMs) - Magnetic field increases the accumulation of MPMs at the target site - Higher toxicity of MPMs to HepG2.2.15 cells than of free drug - IC_50_ is reduced in QCT-loaded MPMs (17.02 µM) compared to the free drug (207.90 µM)	[84]
PLA-PEG	Fe_3_O_4_	DOX		MRI pH-release	- The MPM diameter is SPION concentration dependent - The incorporation of SPIONs significantly increases the drug loading from 3.3% to 12.4%, and drug loading efficiency from 19.8% to 90.9% - *T*_2_ increment in micelles with increased SPION concentration - Prolonged circulation half-live and good stability in vivo	[86]
PCL-*b*-PEG	Fe_3_O_4_	DOX	PBA	optical imaging magnetic drug delivery pH-release	- Higher cell uptake in rod-like MPMs than in the spherical MPMs - Improved DOX delivery and accumulation in the tumor using a dual targeting strategy: actuation of a magnetic field of 0.1 T and active targeting (PBA ligand) - Higher inhibition rate (83%) of tumor growth in rod MPMs with dual targeting - Prolonged circulation half-live (>24 h), and slow blood clearance in rod MPMs with dual targeting	[85]
PLGA-*b*-PEI-*b*-PEG	Fe_3_O_4_	DOX	cRGD	MRI-guided therapy pH-release	- Faster release of DOX at pH 5.3 > pH 6.0 > pH 7.4 - Higher inhibition rate of tumor growth with MPMs (50%) compared to the free drug (20%) - Negligible harmfulness in vivo - Prolonged half-life blood circulation of MPMs (31.2 h) in comparison to the free drug (19.5 h)	[100]
PAsp(DBA-*co*-DIP)-*b*-PEG	Fe_3_O_4_	DOX	-	MRI/optical imaging pH release	- Faster release of DOX at pH 5.0 > pH 7.4 - Very low cytotoxicity to HepG2 cells - Higher survival time (>70 days) with MPMs in >80% of the animals - DiR fluorescence imaging of the tumor tissue	[1]
PCL-*b*-PAELG	Fe_3_O_4_	DOX	Gal/Lac	MRI redox release	- DOX release mediated by GSH (10 mM) - Higher *r*_2_ values (168.1–259.4 mM^−1^ s^−1^) than Feridex^®^ (111.5 mM^−1^ s^−1^)	[83]
PCL-*b*-HA	Fe_3_O_4_	DOX	-	MRI redox release	- DOX release mediated by GSH (10 mM) within 12 h - DOX loading efficiency of 10% and loading content of 11.3–12.5% - Higher cell uptake in HA-SS-PCL than in HA-PCL micelles - Lower cytotoxicity than free drug - *r*_2_ value (221.2 mM^−1^ s^−1^)	[18]
PZLL-g-HA	Fe_3_O_4_	DOX	-	MRI redox release	- DOX release mediated by GSH (10 mM) - DOX loading content of 5.6–6.8% - *r*_2_ value (231 mM^−1^ s^−1^) - suitable as HepG2 tumor targeting nanoprobes	[19]
PEO-*b*-PPO-*b*-PEO (Pluronic F127)	Zn_1.15_Fe_1.85_O_4_	OA-R837	OVA_257-264_	MRI/optical imaging magnetic delivery redox release	- Release of OVA + 2 adjuvants (Zn_1.15_Fe_1.85_O_4_ + OA-R837) - OVA release mediated by GSH (10 mM) - Enhanced delivery of SIM-micelle to lymph node by a magnetic field - in vivo upregulation of TNF-α and IFN-γ, and stronger T cell responses in MPMs actuated by a magnetic field. - 100% survival rate without recurrence for at least 60 days (mice model)	[55]
PAsp(MEA-*co*-DIP)-*b*-PEG	Fe_3_O_4_	SF	Ab_GPC3_	MRI pH and redox release	- SF release mediated by pH 5 > pH 7.4, and GSH (10 mM) - SF loading content of 3.56% - MPMs inhibit tumor growth - *r*_2_ value is 2.5-fold higher in MPMs than in hydrophilic SPIONs	[101]
PCL-*b*-PGA	Fe_3_O_4_	DOX	-	MRI pH and redox release	- DOX loading content of 10.14% - DOX release mediated by pH 5 > pH 7.4, and GSH (2 mM < 10 mM) - Selective toxicity to tumor cells - *r*_2_ value of 192.06 mM^−1^ s^−1^ - 100% survival rate during the treatment with MPMs (50% with free DOX)	[81]
PEG-PU-PCL-PU-PEG	Fe_3_O_4_	DOX	FA	MRI pH- and redox-release	- DOX loading content of 23% - DOX release mediated by pH 6.5 > pH 7.4, and GSH (10 mM) - Higher *r*_2_ value (89.5 mM^−1^ s^−1^) than in SPIONs (54.6 mM^−1^ s^−1^) - Higher inhibitory effects on tumor growth with magnetic field	[89]
(CA)_2_-Lys-(PAsp(DMA))	Fe_3_O_4_	siRNA/ASO	-	MRI tracking of NSCs neuronal differentiation	- Enhanced neuronal differentiation of NSCs in vitro and in vivo - Improved recovery of the damaged tissue after ischemic stroke - Higher *r*_2_ value (674.1 mM^−1^ s^−1^) than free SPIONs (72.16 mM^−1^ s^−1^)	[12]
CAM-HA (PLL coating)	Fe_3_O_4_	plasmid (pLuc)	-	MRI-guided gene delivery	- In vitro magnetofection of MPMs with pLuc plasmid	[33]
OAMAM-*b*-DEX	Fe_3_O_4_	BPD	-	MRI photo release PDT	- BPD loading content of 30% - Higher *r*_2_ value (383 mM^−1^ s^−1^) than SPIONs (Feraheme^®^; 55.26 mM^−1^ s^−1^) - Slower tumor growth in a 4T1 murine model with MPMs + PDT	[23]
PCL-*b*-PEG	Mn_0.6_Zn_0.4_Fe_2_O_4_	-	HA	MRI radiotherapy MHT	- AMF: 178 kHz, 64.1 A, led to a local temperature variation of +7 °C - *r*_2_ value of 331 mM^−1^ s^−1^ - Decreased tumor size with combined MHT and radiotherapy	[54]
C_16_-g-HA	Fe_3_O_4_	docetaxel	-	MRI photo-thermal therapy thermo release	- Docetaxel loading content of 10.9% and encapsulation efficiency of 58.0% - *r*_2_ value of 158.6 mM^−1^ s^−1^ - Cell uptake increased with magnetic targeting (50 mT) - Increased release after irradiation (laser: 808 nm, 10 W cm-2, 10 min)	[20]
PPI-*b*-TEGME	Fe_3_O_4_	DOX	-	MHT thermo release	- DOX loading content of 8.13% and encapsulation efficiency of 55% - Increased release with temperature (37 °C < 45 °C) and with AMF (160 kHz, 328 Oe, 5 min exposure) - Synergistic effect of thermo-chemotherapy in toxicity of Hepa 1-6 cells	[8]
PHEP-*b*-PEG	Fe_3_O_4_ (c)	emodin	-	MRI magnetic targeting MHT thermo release	- *r*_2_ value of 271 mM^−1^ s^−1^ - Emodin encapsulation efficiency of 73.8% - Increased release with temperature (37 °C < 45 °C), and with AMF (35 kA m^−1^, 10 min exposure) - AMF (30 kA, 312 kHz, 10 min) combined with MHT + CHT led to tumor inhibition and prevention of tumor recurrence.	[31]
P(AAm-co-AN)-g-PEG	Fe_3_O_4_	DOX	A54	hyperthermia (microwave) thermo- release	- Microwave: 8 W, 30 min led to a local temperature variation of +13 °C - Increased DOX release with microwave (>43 °C) - Improved tumor accumulation of MPMs with A54 targeting - Anti-tumor efficiency enhanced by microwave hyperthermia	[102]
P(AAm-co-AN)-g-PEG	Fe_3_O_4_	DOX	-	NIR imaging photo-thermal therapy thermo release pH release	- DOX loading content of 8.7% - DOX release mediated by pH 5.5 > pH 6.5 > pH 7.4 - Increased release after irradiation (laser: 808 nm, 2 W cm^−2^, 3 min) - MPMs elevate temperature in the tumor after NIR irradiation (5 min) - Reduced tumor volume after irradiation; damage to tumor cells	[32]
PHEMA-*b*-PEG	Mn_0.6_Zn_0.4_Fe_2_O_4_	Pt(IV)	-	MRI pH and redox release magnetic targeting MHT	- Pt(IV) loading content of 22.5% - Release mediated by pH 5.0 > pH 7.4 (+GSH: 5 µM/1 mM) - MHT improved penetration of MPM in tumors - Higher tumor inhibition with combined magnetic targeting (180 mT) + MHT (114 kHz, 15.9 kA m^−1^, 20 min)	[16]
PAE-*b*-PEG/DPPC	Fe_3_O_4_	DOX	-	MRI/optical/photoacoustic imaging MHT photo-thermal therapy pH- and thermo- release chemodynamic therapy	- DOX loading content of 1.082% - Release mediated by pH 5.0 > pH 6.5 > pH 7.4, and increased with a magnetic field (500 kHz; 20 kA m^−1^) + laser (808 nm + 1 W cm-2; + 17 °C) from 44% to 83% - Magnetic guidance improved MPMs accumulation in tumors - Higher tumor inhibition with MPMs + combinational MHT/CHT/chemodynamic therapy (~94%) in comparison to free drug (54%)	[103]

The full terms are indicated in the abbreviation section.

Another promising molecule for cell targeting is HA. Together with the advantages of using HA as a biomaterial, the functional dysregulation of the two main HA receptors, CD44 and RHAMM, has been associated with chronic inflammation and cancer, providing HA-decorated micelles with features diseased cells recognize more effectively [104]. Cancer cell targeting via HA receptors was investigated using HA-modified PCL-PEG MPMs, where the external HA layer was created to link CD44 in lung cancer [54]. HA-modified MPMs significantly enhanced the MPM intake by A549 cells, a human lung adenocarcinoma cell line, in comparison with non-HA-modified MPMs. The MPMs held cellular selectivity being preferentially internalized by A549 cells relative to normal human bronchial epithelial cells. 

Following a similar strategy, the PCL-*b*-PEG copolymer was end-functionalized with phenylboronic acid (PBA). PBA is a nontoxic, inexpensive, and nonimmunogenic synthetic molecule with high affinity and selectivity to salic acid. The micelles were loaded with DOX and SPIONs and investigated for PBA-mediated endocytosis by salic acid-positive tumor cells. The complementary actions of dual magnetic and active targeting together with the rod-like shape of the - DOX-loaded PBA–MPMs resulted in an 83% inhibition tumor rate in a hepatocarcinoma model [85].

The delivery of immunotherapeutic agents with high spatial and temporal accuracy to disease-relevant tissues constitutes a valuable instrument transversal to immunotherapies, vaccines, and tissue engineering and regenerative medicine (TERM). The internalization of nanocarriers by immune cells is desirable not only for tracking and targeting but also for giving information on pathological conditions that immune cells are naturally attracted to. In this sense, MPMs have been designed for iron delivery to macrophages to empower a sterile inflammatory response in tumor niches, where the activation of macrophages has been correlated with the inhibition of tumor growth. Bauer et al. [82] used the iron from the SPIONs embedded in PCys(SO_2_Et)-*b*-PSar micelles to act as an immune-therapeutic agent. The iron released influenced the iron metabolism and oxidative defense in macrophages, reflecting a robust inflammatory response due to glutathione (GSH)-dependent particle degradation in phagosomal compartments. The inflammatory state caused by MPMs in vivo was further confirmed in the mice lungs after the intra-tracheal instillation of MPMs, which evidenced increased expression of CD80 and the pro-inflammatory cytokines IL-1β and IL-6 after 24 h of treatment, supporting the potential for the activation of macrophages as adjuvant therapy.

On the road to avoiding premature nanotherapeutic clearance from the targeted tissue or aiming at the repair or regeneration of damaged tissues, macrophage activation may not be ideal. Early clearance of MPMs can significantly affect the effectiveness and the off-target toxicity of the payload and, in some cases, the phagocytic mechanisms may also contribute to the deposition of MPMs in the liver, spleen, or kidney during MPM removal. To impart the anti-phagocytosis capability, prevent early clearance, and improve payload efficacy in-situ, Jiang et al. [91] functionalized a self-peptide derived from the glycoprotein CD47 in biodegradable photo-luminescent water-borne polyurethane (BPLP-WPU) micelles. These micelles were loaded with DOX and SPIONs and co-functionalized with a neuropeptide YY1 with high affinity to Y1R, an overexpressed receptor in cancer. The self-peptide modification minimized the opsonization of macrophages to BPLP-WPU micelles and contributed to the SPION accumulation in the tumors of nude mice. Moreover, the modified micelles showed the best inhibition effect on tumor growth, and attained significant biosafety with 100% animal survival 100 days after treatment, along with no evidence of abnormal effects in vital organs [91].

The permanence of MPMs in the body for longer periods may be beneficial for stimulating proper healing and selectively regulating immune functions in pathological conditions without compromising healthy functions. A forerunner work on a bio-functionalized construction was developed by Nguyen et al. [105] using a nanofiber hydrogel for sustained therapeutic delivery and contact guidance aiming at spinal cord injuries. Micellar nanoparticles produced with PCL-PEG and PCL-*block*-poly(2-aminoethyl ethylene phosphate) were complexed with oligonucleotides (miRNA-222) and incorporated in a matrix of PCL-*co*-ethyl ethylene phosphate nanofibers, collagen, and protein neurotrophin-3. This temporary framework supported neural engineering through topographical signals as well as the localized and sustained release of synergistic biomolecules that enhanced axon regeneration, directed neurite extensions, and supported remyelination in nerve injury with good host-implant integration [105]. The inclusion of multimodal loaded MPMs in biological or artificial tissue substitutes combines precision guidance through spatiotemporal control of nanotherapeutics with structural features, which could greatly improve the performance and longevity of implants and assist regenerative medicine.

A less-explored functionality of MPMs is their scavenger potential. Instead of intracellular modulation, magnetic micelles can be used to remove molecules or cells from extracellular environments. MPMs derived from polystyrene (PS)-*b*-DNA with high-density DNA corona and PS-coated SPIONs were produced for bacteria-targeted therapeutics [106]. The MPMs were designed to overcome the expensive and species-specific antibody-based methods by selectively targeting and clearing bacteria based on the Gram classification. The discrimination between Gram-positive and Gram-negative bacteria was demonstrated by a differential receptiveness to cell walls targeting antibiotics, envisioning a timely administration of the most suitable antibiotics in life-threatening situations (e.g., sepsis). The MPMs effectively selected, captured, and concentrated Gram-positive strains from mixed strains in a simple and relatively fast procedure to be applied at the point of care. Likewise, other criteria can be applied to remove molecular inflammatory mediators, metabolites, or regulatory factors feeding damaged or diseased niches.

## 4. Magnetic Micelle Applications

### 4.1. Imaging

MRI is a well-established noninvasive diagnosis tool for obtaining anatomical images with excellent resolution, sensitivity, and tissue penetration depth. In recent years, highly sensitive MRI probes with enhanced signal intensity, biocompatibility, and long blood circulation time have been developed combining molecular imaging and nanobiotechnology. The design of sophisticated probes anticipates desirably visualization, identification, and tracking of biological systems at the cellular and molecular scales supporting optimal detection and therapeutic modalities.

In the last decades, SPION formulations were clinically approved as *T*_2_ contrast agents for MRI (e.g., Feridex IV^®^) and continue to be investigated for cancer imaging and for the detection of inflammatory lesions [47,48]. SPIONs significantly decrease the signal intensity by shortening the hydrogen transverse relaxation time (*T*_2_) causing the darkening of the interfered regions. The incorporation of SPIONs within micelles successfully enhances the MRI signal by increasing the magnetization per carrier, allowing a much more significant response to magnetic fields and thus an enhancement in MRI sensitivity [38,79,86,107].

**Figure 4:**
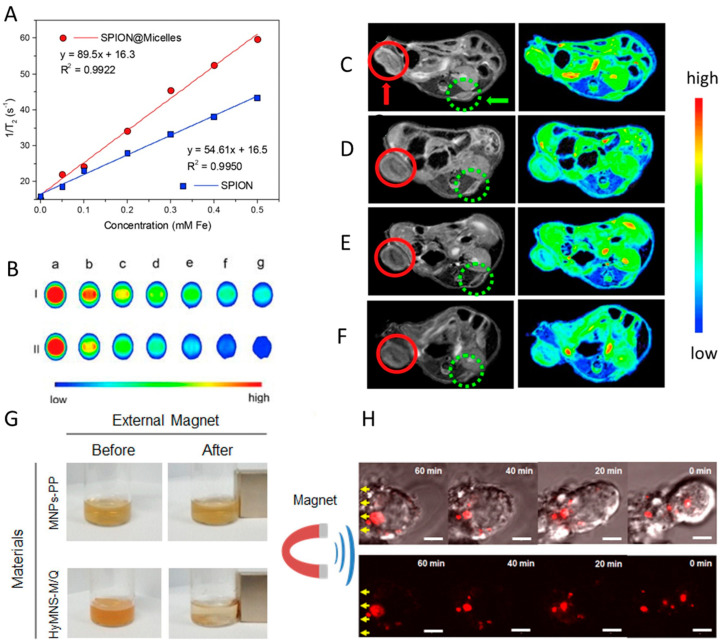
Imaging features of magnetic micelles. *T*_2_-weight MRI images of SPIONs and SPION@micelles in vitro (**A**,**B**) and in vivo (**C**–**F**): (**A**) *T*_2_ relaxation rates as a function of iron (Fe) concentrations; (**B**) *T*_2_-weight MRI images of SPIONs (I) and SPION@ micelles (II) recorded on a 1.5 T clinical MRI instrument at different Fe concentrations (mM): a, 0; b, 0.05; c, 0.1; d, 0.2; e, 0.3; f, 0.4; g, 0.5; (**C**–**F**) In vivo *T*_2_-weighed MR images of HeLa tumor-bearing mice before (**C**) and after intravascular injection of SPION loaded micelles for (**D**) 1 h, (**E**) 3 h and (**F**) 7 h acquired on a 7.0 T MRI instrument. The tumors in the left and right flanks are identified by green dots and red circles, respectively. The magnetic field was applied to the right tumor while the left was not. (**G**) Photos showing the magnetic responses of polymeric micelles incorporating magnetic nanoparticles and quantum dots (HyMNS-M/Q), and magnetic nanoparticles coated with poly(maleic anhydride-alt-1-octadecene)-poly(ethylene glycol) (MNPs-PP) after applying an external magnetic field; (**H**) Real-time image of HyMNS-M/Q migration (yellow arrows) in the cytoplasm of a living cell under the external magnetic stimulus. Scale bar: 3 µm. Adapted with permission from Refs. [38, 89]. Copyright 2022, Elsevier.

In a study by Chu et al. [22], SPIONs incorporated in a chitosan-based micelle enhanced the *r*_2_ relaxivity by 26 times in comparison with individual SPIONs and sustained a high *T*_2_ contrast to the extravascular space, yielding especially detailed images of liver vasculature. These findings are in agreement with the work by Wei et al. [89], in which MPMs built from a multi-block polyurethane incorporating SPIONs presented a higher *r*_2_ (89.5 mM^−1^ s^−1^) than that of SPIONs alone (54.6 mM^−1^ s^−1^) together with a higher efficiency in signal darkening owing to the clustering of SPIONs in the micellar core (Figure 4A,B).

To confirm the MPM imaging potential, nude mice were inoculated with HeLa cells to generate two tumors in the hind leg region. MPMs were intravenously injected every 3 days for 12 days, and the right tumor was 1 h exposed to an external magnetic field immediately after the injection. The *T*_2_ images (Figure 4C–F) showed a signal darkening of both tumors over time. Nevertheless, the right tumor revealed a much faster and higher degree of signal darkening demonstrating the potential of magnetically guided MRI.

Park et al. [38] also showed that levan MPMs exhibit an increase in the *r*_2_ relaxivity of ~45% over SPIONs alone. The liver of mice treated with intravenous MPMs revealed a 54% increase in the change in the signal-to-noise ratio, encouraging MPMs as worthwhile MRI probes. The authors further explored the co-incorporation of SPIONs and gold nanoparticles as well as SPIONs and quantum dots for magneto-fluorescent imaging. (Figure 4G,H). The levan micelles were magnetically manipulated in living cells while being optically tracked, serving as a successful dual-mode imaging probe in vivo.

In consideration of the multimodal imaging potential, MPMs have also been studied in the format of magneto-telodendrimers, envisioning the targeted therapy of lymph node metastasis [79]. By controlling the particle dimensions and by loading Nile Red into the core of the micelle, MPMs can evaluate the macrophage uptake efficiency and investigate the feasibility of MRI bimodal lymphography in vitro [79].

The morphology of MPMs has been described as influencing the signal intensity, and thus the visibility, of internal body structures on MRI. Zou et al. [90] produced magnetic worm-like micelles using SPIONs and poly(4-vinylpyridine)-*block*-polyethylene glycol (P4VP-*b*-PEG) followed by a DNA co-assembly to induce the linear aggregation and fusion of spherical micelles. The large number of SPIONs in the linear core of magnetic worm-like micelles led to an ultrahigh *r*_2_ about 6.6 times that of spherical micelles, and 20 times that of individual SPIONs, resulting in a highly sensitive MRI probe. Despite a length of approximately 536 nm, the small width (20.5 nm) of worm-like MPMs enabled a longer blood circulation time in comparison to free-SPIONs.

From a treatment perspective, tracking transplanted cells is essential for ensuring the safety and efficacy of the therapeutics and understanding implanted cell dynamics and regeneration potential [75]. MPMs made of SPIONs and cholic acid-lysine-poly(aspartic acid-dimethylethanediamine) ((CA)_2_-Lys-PAsp(DMA)) were produced to overcome the sub-optimal cellular uptake of free SPIONs with simultaneous visualization of implanted cells. The labeling of neural stem cells (NSCs) with MPMs did not affect their proliferation and neuronal differentiation in vitro, after labeled cells injection intracerebrally. MPM allowed a high imaging sensitivity and clear tracking. This study demonstrated the feasibility and safety of these cationic MPMs for convenient high payload cell labeling with robust biocompatibility and high MRI sensitivity [75].

### 4.2. Delivery and Release of Therapeutics

#### 4.2.1. Magnetic Targeting

Polymeric micelles (PMs) hold promise for the delivery of therapeutic agents including hydrophobic medicines and protecting from biological interactions and biodegradation in vivo. Furthermore, PMs have shown selective accumulation and enhanced bioavailability of incorporated drugs compared with free-drug formulations, since Genexol^®^, a PM made of methoxypoly(ethylene glycol)-*block*-poly(D,L-lactide) containing PTX, reached the market for cancer treatment [108]. Despite the significant progress in the field, the targeting efficacy can be potentiated by the magnetic responsiveness of MPMs for generating precise and remotely controlled payloads, ensuring a high therapeutic concentration locally, and avoiding adverse side effects associated with traditional drug delivery routes. 

Magnetic targeting, also referred to as magnetophoresis, can be achieved using stationary or time-varying magnetic field gradients. While stationary magnetic fields are mainly applied to accumulate the nanocarriers at a particular region, for instance, a tumor, the time-variant magnetic fields are pursued to stimulate thermal treatments (hyperthermia or thermal ablation) and to induce payload release in a controlled and desirable manner.

Magnetic drug targeting is still of great interest for drugs with short half-lives (≤4 h), for reducing drug dosages and frequency of administration, and for lowering the intensity of undesired effects due to fluctuations in drug concentrations.

The pledge of MPMs for targeted and controlled delivery has already been explored for the central nervous system and cancer treatment [18,109]. To overcome the blood-brain barrier and target delivery of medicines with limited brain entry, MPMs were prepared with poly(carboxyl-ε-caprolactone-*block*-poly(ethylene glycol)) (PCCL-*b*-PEG), SPIONs, and NPX. MPMs were injected into rats (10 mg kg^−1^) followed by the actuation of a 0.4 T magnetic field on the animal skull. MPMs showed a prolonged circulation time in blood compared with the free drug group, and accumulated in the brain, especially in the group treated with smaller MPMs (<150 nm), suggesting an efficacious transportation and deposition mediated by magnetic guidance [76].

Envisioning targeted cancer therapies, dasatinib (DAS) was incorporated in zein-LF MPM. In this strategy, magnetic guidance was combined with active targeting of LF corona to cancer cells via LF receptor-mediated endocytosis. Magnetically stimulated (0.5 T) MPM significantly enhanced a cytotoxic effect on breast cancer cells, promoting a valuable anti-tumor effect at lower doses [24].

Together with the payload delivery, the loading profile can be controlled and improved by magnetic field action. The incorporation of SPIONs in micellar systems of PLA-PEG significantly increased the content and loading efficiency of DOX [86]. The loading of DOX increased from 3.3  ±  2.0% to 12.4% when 15% wt SPIONs were co-loaded in the micelles. Moreover, the SPION-loaded micelles showed much higher drug loading efficiency (90.9%) than the SPION-free micelles (19.8  ±  2.0%), supporting the use of MPMs for more efficient theranostic applications.

Since the accumulation and payload release from magnetic nanocarriers can be selectively controlled, the therapeutic index of a bioactive molecule can be locally augmented by increasing its concentration at the region of interest minimizing the effects on off-target tissues and their potential complications [110].

#### 4.2.2. Thermal Stimulation

Magnetic stimulation has further been employed to increase the accumulation of MNPs in tumor cells, leading to higher magnetic hyperthermia potency and targeted local heating treatment. When used as hyperthermia agents and thermal triggered vehicles, MPMs are subjected to different energy sources from microwave to NIR radiation or magnetic fields. These stimuli induce motion in the magnetic dipoles in MNPs that in turn results in heat dissipation, provoking drug release and diseased cell death.

**Figure 5:**
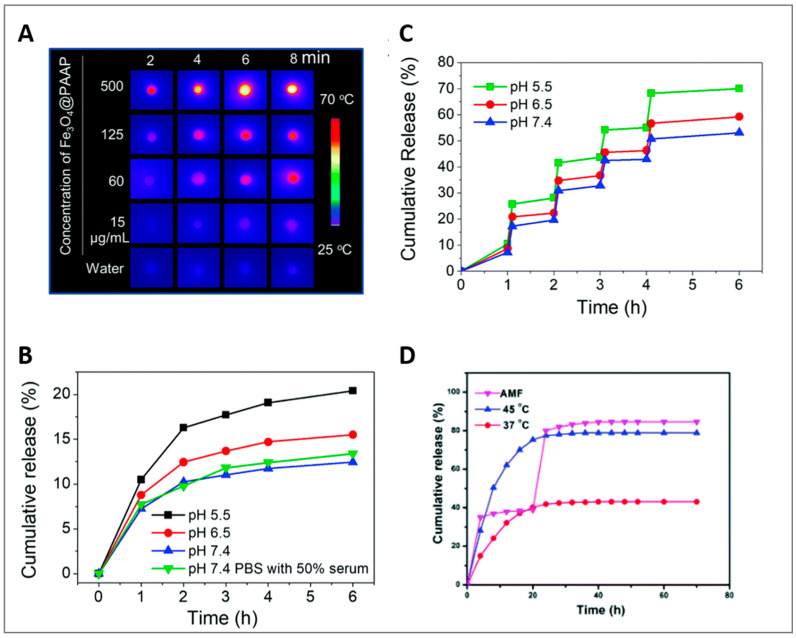
The multi-responsive potential of magnetic polymeric micelles. (A-C) DOX-Fe_3_O_4_@PAAP micelles produced from P(AAm-co-AN)-g-PEG, SPIONs and DOX, evidenced a pH and NIR irradiation controlled release of DOX: (**A**) Thermographic images of Fe_3_O_4_@PAAP micelles after exposure to NIR radiation (808 nm laser at 2 W cm^−2^); (**B**) Cumulative release curves of DOX in PBS (pH 5.5, 6.5 and 7.4) with or without serum at pH 7.4; (**C**) Cumulative release curves of DOX in PBS (pH 5.5, 6.5, and 7.4) upon irradiation with a NIR laser for 3 min. (**D**) Cumulative release curves of DOX from PPI-*b*-TEGME, SPIONs and DOX micelles at 37 °C, 45 °C, and after a 5 min exposure to an AMF. Adapted with permission from Refs. [8, 32]. Copyright 2022, The Royal Society of Chemistry.

Li et al. [102] investigated a microwave-activated thermo-chemotherapy approach in a liver tumor using MPMs assembled from P(AAm-co-AN)-g-PEG, SPIONs, and DOX. The presence of SPIONs enabled elevating the tumor temperature by 13 °C using mild microwave (8 W) in comparison to the increment of only 6 °C without SPIONs. The release of DOX was improved in MPM from 10 to 50% above 43 °C, which stands for the upper critical solution temperature (UCST) of the micellar system [111]. When the thermo-chemotherapy was triggered by NIR laser irradiation [32], the photo-thermal effect increased with irradiation time and MPM concentration (Figure 5A). Without NIR irradiation, the release of DOX gradually increased as the pH decreased, with the cumulative release percentages being 21.28%, 24.96%, and 33.18%, respectively for pH 7.4, 6.5, and 5.0. After 3 min irradiation, the cumulative release boosted from 10.5% to 25.8% at pH 5.5, from 8.78% to 20.9% at pH 6.5 and from 7.22% to 17.32% at pH 7.4 (Figure 5B,C). After 6 h and at pH 6.5, about 60% of DOX was released in comparison with the 16% observed without NIR irradiation, indicating the drug release was triggered by the photo-thermal heating of SPIONs via the MPM disassembly at 43 °C.

Local magnetic hyperthermia (MHT) under AMF is a contactless and promising therapy influenced by the heating efficiency of MNPs. The heating efficiency is tuned by the amplitude and frequency of the applied magnetic field as well as the structural and magnetic properties of MNPs. Compared with photo-thermal heating using NIR radiation, MHT performs by remotely controlling the heating effect without penetration-depth limit, being particularly useful for the treatment of nonsuperficial pathologies (e.g., tumors). Moreover, the incorporation of MNPs into thermo-responsive MPMs opens up the possibility for AMF-mediated control of the drug release. The combination of AMF with MNPs favors a localized temperature increment with the benefit to be gathered with chemo- or radio-therapies for synergistic action and, consequently, with improved therapeutic effects. Using an MHT approach, MPMs self-assembled from PPI-*b*-TEGME, SPIONs, and DOX were investigated as anti-cancer drug carriers [8]. While the cumulative release of DOX reached about 40% at 37 °C when the temperature was elevated to 45 °C the rate of drug release was quicker reaching 80% of cumulative release. This incremental value is explained by the thermal disintegration of the MPM above the LCST, in this case, 41 °C (Figure 5D). When the AMF was applied for 5 min, the drug release reached 35% within 1 h, and 80% after 24 h and an additional 5 min exposure. Likewise, the removal of the AMF resulted in a very low drug release. MPMs were further administered to Hepa 1–6 cells and exposed to a 20 min AMF treatment (328 Oe, 160 kHz). The combination of MHT and DOX had a negative impact on cell proliferation and survival evidencing an efficient thermo-chemotherapy performance.

#### 4.2.3. MPM Responsiveness to Extracellular and Intracellular Stimuli

Beyond the magnetic responsiveness, smart magnetic micelles have been chemically modified with moieties to expand their on-demand drug release to different cell environments (e.g., pH change, altered redox potential) creating exciting opportunities for precision and multimodal nanoplatforms to modulate molecular and cellular events. In diseased cancer cells, GSH is present in concentrations 100 to 1000 times higher than those available in extracellular fluids and circulation and was shown to efficiently cleave disulfide links in amphiphilic copolymers [112]. In a work by Yang et al., MPMs were produced using PCL-*b*-PAELG with a disulfide link between the blocks that was established to empower the redox responsiveness, targeting GSH [83]. PCL-*b*-PAELG-based MPMs exposed to intracellular levels of GSH (10 mM, pH7.4) accelerated and prolonged the DOX release profiles [83]. Furthermore, cell studies with HepG2 cells revealed that DOX-free MPM have excellent biocompatibility but in the presence of DOX-loaded MPMs, the viability of HepG2 cells decreases as the DOX concentration increases evidencing suitable therapeutic effects. The loading of DOX in the MPM induced lower cytotoxicity against HepG2 cells in comparison with free-DOX, suggesting a potential reduction of side effects to non-target tissues.

A wide range of studies have been published on redox-responsive MPM using different disulfide-linked copolymers, namely, PCL-*b*-PEG [97], PZLL-g-HA [19], PCL-*b*-HA [18], or by core disulfide cross-linked cores, for instance, resourcing to PCys(SO_2_Et)-*b*-PSar [82] for increased chemotherapeutic value with cytoplasmic GSH.

The design of pH-sensitive MPMs is also being pursued in cancer treatment. The structural stability of a pH-responsive micelle in the bloodstream (pH 7.4) can be rapidly disrupted after reaching the targeted tissue and their chemotherapeutic payload released in response to the tumor acidic extracellular environment (pH ~6.5) or the lysosomal compartments of cancer cells (pH ~5.5) [1]. MPMs have been investigated using different materials, including octyl-chitosan derivatives [22], PCL-*b*-PEG [85], PLGA-*b*-PEI-*b*-PEG [100], zein-LF [24], and a combination of PLA-PEG and PLA-CHI-Spm (PLA-PEG/PLA-CHI-Spm) [98]. Polymer chemistry resourcing to specific modifications, for instance, the inclusion of an acid-sensitive ligand in a polyurethane polymer [91], the protonation of chemical groups [92], or the grafting of pH-sensitive groups [1], encourage a faster pH-dependent release of the therapeutic cargos. Liu et al. [92] proposed an MPM assembled from PCCL-*b*-PEG incorporating PTX in the core. The MPM allowed a faster drug release at pH 6.5 than at pH 7.4, in response to the protonation of carboxylic groups in PCL and the protonation of PEG. The decrease in pH breaks the hydrogen bonds between carboxylic groups and increases the rigidity of PEG caused by the loss of hydrogen bonds between water molecules and PEG, thus facilitating pH-responsive payload release. A pH-sensitive copolymer developed by grafting the pH-sensitive groups *N*,*N*-diisopropylamino ethylamine (DIP) and *N*,*N*-dibutalamino ethylamine (DBA) to the poly(β-benzyl L-aspartate) (PBLA) segment of PBLA-PEG copolymer was assembled into MPM and combined with SPIONs and DOX [1]. The MPMs endorsed a quicker drug release at pH 5.0 in comparison with physiological pH, owing to the protonation of more amino groups at pH 5.0, which turned the hydrophobic segment into hydrophilic, inducing core swelling and DOX release. The functionality of this elegant anti-cancer system was further validated in nude mice bearing subcutaneous HepG2 xenografts. Injected PBLA-PEG MPMs were shown to provide significant contributions to the inhibition of tumor growth and low side effects in hepatoma treatment.

Redox and pH dual-sensitive MPMs have been also reported in the literature. Such a multimodal approach intends to rapidly release drugs in cancer cells and tumors, considering both the acidic environment and the richer GSH levels in diseased niches [89,101]. MPMs were constructed from poly(*N*-(2-aminoethanethiol-*co*-2-aminoethyldiisopropylamine)aspartamide)-*block*-poly(ethylene glycol) (PAsp(MEA-*co*-DIP)-PEG), a copolymer functionalized with DIP and 2-aminoethanethiol (MEA) for pH and redox sensitivity, respectively [101]. The micelles were loaded with SPIONs and sorafenib (SF), a kinase inhibitor approved for the treatment of multiple cancers. SF release increased from 10% at pH 7.4 to 30% at pH 5.0 given DIP protonation that guided the hydrophobic-to-hydrophilic change of PAsp(DIP). Additionally, in the presence of GSH, the release profile was accelerated, reaching ~55% and 86% at pH 7.4 and pH 5.0, respectively, in 24 h. An HepG2 tumor in mice confirmed the in vivo therapeutic potential of PAsp(MEA-*co*-DIP)-PEG MPMs.

Overall, these encouraging outcomes bring attention to the skillfulness of MPMs as effective stimuli-responsive transporters for improved targeted therapeutics.

### 4.3. Oligonucleotides Delivery

Oligonucleotide-based technologies have received significant interest as nanotherapeutics for various diseases. Oligonucleotides accommodate antisense oligonucleotides, small-interfering RNAs, microRNA moieties, and DNAzymes/ribozyme with pivotal roles in the regulation of key proteins in biological processes, for instance, cellular organization, cell fate, cell phenotype, proliferation, apoptosis, or tissue homeostasis and maintenance. Unlike conventional small-molecule drugs, a single oligonucleotide can target a range of pathological features, and thus be an effective alternative to drug-based therapies. The approval by the FDA of oligonucleotides for the treatment of neurodegenerative and respiratory disorders, cancer, and diabetic retinopathy has opened new avenues for using these nanomachines to treat and manage a wide range of pathologies on account of their customizable design to selectively target any gene with minimal or predictable off-target effects.

Magnetofection involves the transfection of magnetic vectors into cells forced by a static magnetic field and is becoming a routine technique in gene therapy for the internalization of RNA species and plasmids, benefiting from a higher degree of cell penetration relative to non-magnetic strategies. The transfection efficiency can be significantly increased by binding electrostatically cationic polymeric MNPs to the negatively charged phosphate of the genetic material forming stable magnetic complexes that are internalized under the guidance of a magnetic field. In this regard, micelles comprising a cationic hydrophilic block such as PEI or CHI hold promise as non-viral vectors for oligonucleotide delivery featuring target delivery control with cell tracking [113]. In comparison to conventional vectors, cationic micelles enhanced the complex’s stability, protect oligonucleotide from degradation, enhance cellular uptake and improve intracellular trafficking via an endosomal escape mechanism [114].

Naturally occurring nucleotides are rapidly degraded in vivo, which makes them a translational challenge. To improve their pharmacokinetics and circulating half-lives, chemically modified nucleotide analogs against nuclease degradation are normally approached although they can induce unwanted bioactivity and toxicity, posing major limitations to efficient oligonucleotide distribution. Most of the oligonucleotides approved by the FDA have been administered locally, nonetheless pioneer works on MPMs have been reported to facilitate oligonucleotide bioavailability, lending solutions for crossing biological barriers and transmembrane delivery [33].

SPIONs encapsulated in hydrophobically modified HA micelles have been researched for gene delivery followed by a coating with polylysine for the electrostatic complexation with a plasmid DNA (pLuc). The micelles could safely and efficiently transfer pLuc into fibroblast cells and colon cancer cells from mice. The pLuc transfection was remotely controlled by magnetic fields and the presence of SPIONs capacitated these micelles for MRI-guided gene therapies [33].

Other oligonucleotide-loaded MPMs were projected to guide cell phenotypes and improve recovery after injury. In a work by Lin et al. [12], MPMs were explored for tracking and differentiating therapeutic NSCs since the poor neuronal differentiation of transplanted NSCs still represents a significant drawback in the treatment of ischemia stroke, a leading cause of death and disability in the world [75]. MPMs made of (CA)_2_-Lys-(PAsp(DMA)) and SPIONs were developed to deliver small interfering RNA/antisense nucleotides (siRNA/ASO) into NSCs guiding their differentiation into functional neurons [12]. NSCs were transfected with the siRNA/ASO-loaded micelles aimed to silence the *Pnky* lncRNA, an inhibitor of neural differentiation of NSCs, and injected into the lesion. The spatial and temporal migration patterns of transfected-NSCs were MRI tracked due to the high *r*_2_ relaxivity of SPIONs. The siRNA/ASO-loaded micelles enhanced the percentage of differentiated neurons at 2 and 4 weeks after intracerebral transplantation, and the mice treated with the siRNA/ASO-loaded micelles showed better structural and functional recovery from stroke [12]. The successful outcomes in this work inspire MPMs usage in other injury models emphasizing MPM-assisted technologies for tissue functional recovery with contributions to the identification of the lesion extension or of the inflammatory stage of the damaged tissue. 

### 4.4. Theranostics

The concept of theranostics has roots in nuclear medicine. However, since the 1990s, it has been used in precision and tailored medicine to assist with diagnostic procedures and targeted drug therapy allied toward the monitoring of treatments in order to increase drug efficacy and safety. Personalized medicine, pharmacogenomics, and molecular imaging constitute important pillars of theranostic strategies aiming to assist competent new targeted therapies with adequate benefit/risk to patients, improved drug selection, and a better understanding of molecular, cellular, or tissue information related to pathological conditions.

MPMs are theranostic by nature, promoting a smooth transition from conventional to personalized and precision medicine. MNPs naturally support the noninvasive monitoring and diagnosis potential, whereas the micellar architecture assists with the incorporation of therapeutic agents to deliver and treat.

**Figure 6:**
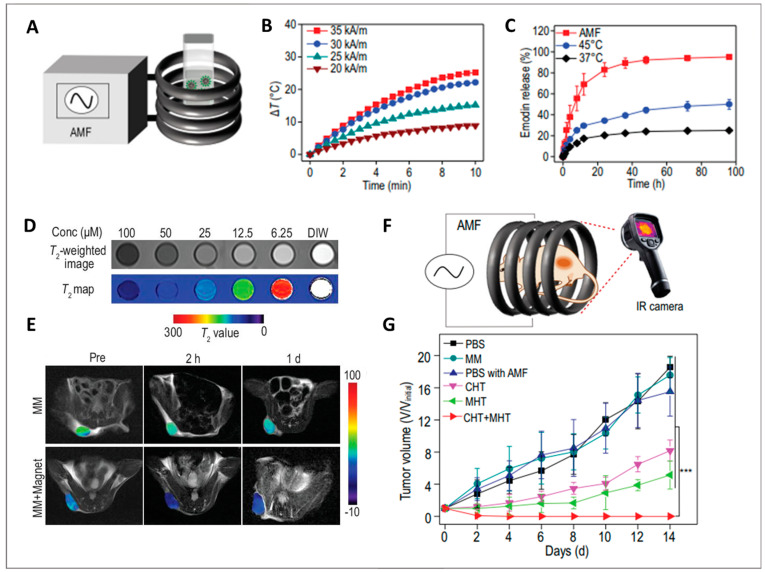
Magnetic polymeric micelles for hyperthermia treatment. (**A**) Schematic illustration of the magnetic hyperthermia in vitro assay; (**B**) Time-dependent temperature curves with different AMF intensities; (**C**) The release curves of emodin from emodin-magnetic micelles (EMM) after a 10 min treatment at 37 °C and 45 °C, and in response to an AMF; (**D**) *T*_2_-weighted image map of EMMs; (**E**) The in vivo *T*_2_-weighted images of the tumor after intravenous injection of non-loaded emodin-magnetic micelles (MM) in the absence and presence (MM + Magnet) of magnetic targeting; (**F**) Schematic illustration of EMM mediated magnetic hyperthermia (MHT) and chemotherapy (CHT) in vivo study with 4T1 tumor-bearing mice; (**G**) The tumor growth curves (statistical analyses were performed using Student’s *t*-test; ∗∗∗ P < 0.001). Adapted from reference [31].

Motivated by the synergistic anticancer action of MHT and chemotherapy (CHT) together with the imaging capability, MPMs have been assembled with poly(2-hexoxy-2-oxo-1,3,2-dioxaphospholane)- *block*-poly(ethylene glycol) (PHEP-*b*-PEG) to load cubic SPIONs and the drug emodin (Figure 6) [31].

The magnetically induced heating increased with both MPM concentration and AMF intensity, with emphasis on the local temperature increment by 24 °C in the presence of MPMs (200 µg mL^−1^) and after a 10 min application of 35 kA m^−1^ field intensity (Figure 6A,B) causing an almost complete release of emodin (Figure 6C). Additionally, the magnetic targeting efficiency of MPMs enhanced the combined MHT and CHT therapeutic effect, with an additional 10% reduction in the viability of 4T1 cancer cells, even at extremely low dosages (10 μg Fe mL^−1^). In mice tumors, an enhanced accumulation of MPM with magnetic treatment was accompanied by a markedly decrease in the *T*_2_ signal intensity which was still detected 24 h post MPM injection (Figure 6D,E). PEG-*b*-PHEP MPMs presented an enhanced permeability and retention effect supporting magnetic targeting guidance into specific body regions. The exposure of MPM-treated mice to a 10 min AMF (30 kA m^−1^, 312 kHz) using a magnetic coil supported the inhibition of tumor growth and prevention of tumor recurrence (Figure 6F,G).

Photodynamic therapy (PDT) is a noninvasive and FDA-approved targeted approach for cancer treatment. Photosensitizers are combined with non-ionizing light generating singlet oxygen that kills irradiated cells. However, photosensitizers such as benzoporphyrin derivatives (BPD) have limited solubility and pharmacokinetics, which can be overcome with MPMs. An MPM composed of OAMAM-*b*-DEX and BPD incorporating SPIONs was assembled to bundle imaging capabilities, photosensitizer localization, and insights into the pathological process [23]. BPD was covalently bonded to DEX using a facile and reproducible synthesis avoiding the premature release of the photosensitizer during systemic circulation. The MPM demonstrated high BPD loading capacity (30%, *w*/*w*) and the incorporation of SPIONs increased the MRI contrast within tumors as a consequence of the MPM accumulation 24 h after intravenous injection in mice. PDT ameliorated anticancer efficacy compared to that of free BPD assessed for up to 22 days after MPM administration. Despite the convenience and suitability for improving tumor imaging before PDT, the predictive value of such strategies largely depends on the type of cancer and of treatment demanding personalized MPM-based constructions addressing the specific features of oncological pathologies.

An example of combinatorial modality is the work by Liu et al. [103], in which MPM made of PEG-PAE and DPPC, DOX and SPIONs approached chemotherapy/hyperthermia/chemodynamic therapy. The DOX release increased with decreasing pH from 18% at pH 7.4, to 33% at pH 6.5, and to 44% at pH 5.5. Moreover, the synergistic effect of NIR laser irradiation (photothermal therapy) and external AMF (MHT) increased the thermal release from ~44% to ~83%, resulting in direct tumor damage. These MPM enabled the conversion of internal hydrogen peroxide, which is typically increased in tumor microenvironment, into toxic hydroxyl radicals (^•^OH) to destroy tumor cells via Fenton or Fenton-like reactions. The combinational therapies led to the most significant tumor inhibition effect (~94%) in comparison with free DOX (54%). Such strategy also supports multiple bioimaging modalities namely magnetic resonance/fluorescence/photoacoustic imaging to monitor the MPM accumulation at the tumor site by means of magnetic targeting and envisioned MRI guided treatment.

The versatility of MPMs has also been applied in studies aiming at adjuvant therapies. Adjuvant therapies are often used after primary treatments (e.g., surgery) and rely on therapeutic agents that enhance the effect of a drug, treatment, or biological system. The suboptimal co-delivery of antigens and adjuvants for immunotherapies was investigated by Li et al. [55] with the construction of sophisticated MPMs incorporating the amine-containing ovalbumin model antigen (OVA_257__–__264_), and using oleic acid-modified imiquimod (OA-R837) and MNPs as adjuvants. In these cancer vaccines, the MPM was formed by pluronic F127 block copolymer modified with a redox-responsive self-immolative linker that also allows the grafting of OVA_257__–__264_ on the MPM surface. OVA_257–264_ is known to stimulate immune cells and supports the measurement of peptide epitope specificity and IFN-γ releasing cells. The OA-R837 and Zn_1.15_Fe_1.85_O_4_ nanoparticles were incorporated into the MPM core to render dual adjuvant action. The release of OA-R837 was mediated by disulfide bond cleavage via intracellular GSH while MNPs magnetically drove the transport of MPM to the lymph nodes to be taken up by immune cells. The magnetic responsiveness of MNPs guided the migration of dendritic cells in vitro, whose increment was proportional to the zinc concentration investigated. In vivo, the strong MRI signal enabled MPMs monitoring and delivery to the inguinal lymph nodes of C57BL/6 mice by an external magnetic field. This multifunctional system successfully enhanced the cancer immunogenicity inhibiting tumor growth with a survival rate of 100% and without evidence of tumor recurrence for at least 60 days. This original study identifies new options for MPMs envisioning immunotherapies, and contributing to the next generation of nanovaccines.

## 5. Conclusions and Future Perspectives

Smart magnetic micelles are burgeoning for improved diagnosis, localized therapeutics, and theranostic applications with promising interventions for infectious diseases, cancer, autoimmunity, and implant rejection. Unlike other artificial vesicles, MPMs are easily and inexpensively synthesized complemented by a multifaceted nature contained in a single structure. The tailor-made physico-chemical properties result in MPMs’ being highly versatile in design and applicability. However, the huge variability regarding the biomaterials, surface engineering, physico-chemical cues, and therapeutic agents used to fabricate MPMs makes it challenging to compare them among studies, leading to contradictory outcomes. 

The impact of MNPs on MPMs’ success is indisputable. Furthermore, MNP’s responsiveness to magnetic fields (e.g., magnetic- targeting, hyperthermia, and drug delivery), and to different energies offer a panoply of new possibilities to enhance imaging and therapeutic effects of MPMs approaching combinatorial modalities in order to minimize side effects and to act selectively. In spite of the very promising reports and in vivo studies, MPM formulations have not been evaluated in clinical trials or in human-oriented procedures. This is particularly intriguing considering that some SPIONs are *T*_2_ contrast imaging agents in clinical use for 20 years and that nonmagnetic polymeric micelles have already been approved by regulatory agencies. It is possible that MPMs have been hindered by the limited mechanistic studies on the long-term influence of MPM formulations in cells and by the paucity of proper models for predicting patient-to-patient variations including nanomaterials’ sensitivity. Likewise, the extreme prudence and hindmost refinements on biosafety and biofunctionality of MPMs together with a lack of standardized guidelines for their evaluation delay the successful commercialization of MPM products, including micellar nanomedicines with smart functionalities oriented to drug delivery platforms and cell-based therapies.

Despite these hurdles, MPMs are emerging as a powerful tool for noninvasive diagnosis, enabling the traceability and monitoring of therapeutic cells or medicines. MPMs can overcome the limited sensitivity and time of acquisition of MRI, the poor tissue penetration of NIR imaging, and the limited availability of photoacoustic-based probes in a single imaging design. The design of nano-sized multimodal probes with accurate and reliable information on complementary imaging data is highly desirable for a precise and fast diagnosis and prevention of diseases (e.g., tumor detection and metastasis monitoring).

Nanomicellar platforms holding magnetic power have shown encouraging outcomes for tissue-targeting low-dosage medicines, anticipating the integration of multimodal imaging MPMs with personalized nanomedicines. Since MPMs can transpose physiological barriers, such as cell membranes and the blood-brain barrier, these nanocarriers can reach difficult-to-access organs and solve the problem of the local delivery of poorly soluble and/or toxic compounds, enhance pharmacokinetic profiles with a superior safety profile for therapeutic drugs, and provide new functions or targets to drug molecules contributing to drug discovery and development.

The introduction of MPMs to immunotherapies and cancer vaccines, in which off-target delivery could detrimentally affect the patient’s health, will likely shape the distribution of immunotherapeutics to the disease site or into disease-relevant tissues with high spatial and temporal accuracy. The continuous innovation in targeted delivery of multiple therapeutics as well as the monitoring and stimulation of desirable cell/tissue responses will boost the real-time screening and identification of pathophysiological contributors enlarging the efficacy of future treatments and aspiring noninvasive clinical solutions.

In a near future, MPMs may have additional functionalities to favor dialogue between artificial vehicles and immune cells. While in vaccine technology it is desirable to elicit immunogenicity, from a TERM perspective, the development of systems that act as promoters of immunological tolerance is highly desired, aiming at disrupting persistent inflammatory signals and facilitating the integration of implants and grafts. In this context, MPMs could be applied directly to damaged sites as therapeutic units directing stem cell differentiation or the polarization of immune cells, preventing chronic inflammatory states and assuring tissue regeneration. The integration of MPMs into tridimensional tissue substitutes (e.g., hydrogels or scaffolds) could also assist implant-host communication and advance healing outcomes at different levels. As part of an artificial tissue, MPMs could increase the efficacy and bioavailability of the MPM-loaded therapeutics locally and enable a spatial and temporal gradient of MPMs following the implant degradation rate whereas tracking and monitoring the regenerative process. In nanocomposite hydrogels, MPMs could also engage in functional soft robotics or biosensing devices to execute programmable complex actions controlled by external magnetic stimuli.

The leverage of immune cells behavior and biomaterial in tissue integration would greatly set a revolution in the field of tissue regeneration and disease management with an outstanding impact on the quality of life of patients. Ultimately, the routine application and clinical practice of MPM-based theranostics could result in more effective and personalized treatments, and significant drug cost savings for the health care system.

New developments in MPMs will unveil innumerous opportunities for holistic MPM nanoplatforms from fundamental biological studies to the diagnosis, prevention, and management of diseases, supporting the next generation of high-performance theranostic agents.

## Figures and Tables

**Table 1:** Building block materials for the hydrophilic and hydrophobic segments of magnetic polymeric micelles.

Hydrophilic Segment (Shell)	Hydrophobic Segment (Core)	
**Synthetic polymers**	**Synthetic polymers**	
- PEG - PGA - PAELG - PAA - PEI - PAsp - PNIPAM - PSar - TEGME - Spm	- PCL - PLA - PLGA - P4VP - PS - PPO - PAsp - PZLL	- PHEP - P(AAm-co-AN) - PPI - OAMAM - PCys(SO2Et) - PAE - PHEMA
**Natural polymers**	**Natural polymers/molecules**	
- hyaluronic acid - chitosan - dextran - lactoferrin - nucleic acids (DNA)	- alkyl groups (octyl, palmitoyl) - bile acids (CA, CAM) - phospholipids (DSPE, DPPC) - zein	

The full designation of the polymers/molecules is indicated in the abbreviation section.

**Table 2:** Self-assembly architectures of amphiphilic copolymers to produce magnetic micelles.

Co-Polymer Type	Examples of Co-Polymer		Micelle
Di-block			
**A-B** 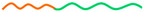	PCL-*b*-PEG PCL-*b*-PGA PCL-*b*-HA PCL-*b*-PAELG PLA-*b*-PEG P4VP-*b*-PEG DSPE-*b*-PEG	PHEP-*b*-PEG PAsp(DBA-*co*-DIP)-*b*-PEG PAsp(MEA-*co*-DIP)-*b*-PEG PAE-*b*-PEG PCys(SO_2_Et)-*b*-PSar PHEMA-*b*-PEG PS-*b*-DNA	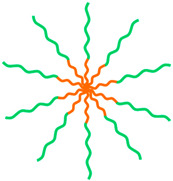
Tri-block			
**A-B-A** 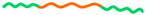	PEO-*b*-PPO-*b*-PEO (Pluronic F127) PNIPAM-*b*-PCL-*b*-PNIPAM		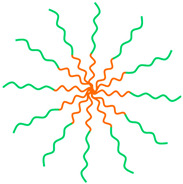
**A-B-C** 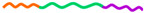	PS-*b*-PAA-*b*-PEG PLGA-*b*-PEI-*b*-PEG		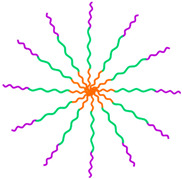
Star-like			
**A_2_B_3_** 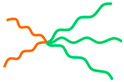	PLGA-PEG		* 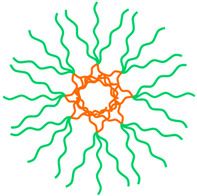 *
Graft			
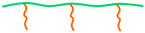	C_16_-g-HA P(AAm-co-AN)-g-PEG PZLL-g-HA Octyl-g-HTCC Octyl-g-PEG-HTCC GCPQ CAM-g-HA PLA-g-CHI-g-Spm		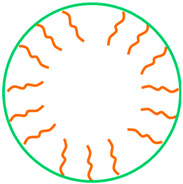
Telodendrimer			
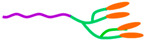	(CA)_4_-Lys_3_-PEG (CA)_2_-Lys-(PAsp(DMA)) OAMAM-*b*-DEX		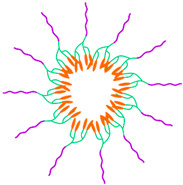

The full designation of the co-polymers is indicated in the abbreviation section.

## Abbreviations

Ab_GPC3_: anti-glypican-3 antibody; AMF: alternate magnetic field; bFGF: (basic) fibroblast growth factor; BPD: benzoporphyrin derivative; BPLP-WPU: biodegradable photo-luminescent water-borne polyurethane; CA: cholic acid; (CA)_2_-Lys-(PAsp(DMA)): cholic acid-lysine-poly(aspartic acid-dimethylethanediamine); (CA)_4_-Lys_3_-PEG: telodendrimer dendritic oligo-cholic acid-*block*-poly(ethyleneglycol); CAM: aminoethyl 5β-cholanomide; CAM-g-HA: aminoethyl 5β-cholanomide-grafted-hyaluronan; C_16_-g-HA: hexyldecyl-*grafted*-hyaluronan; CHI: chitosan; CHT: chemotherapy; CMC: critical micelle concentration; CM-DEX: carboxymethyldextran; DAMPs: damage-associated molecular pattern; DAS: dasatinib; DBA: *N*,*N*-dibutalamino ethylamine; DC: dibucaine hydrochloride; DEX: dextran; DIP: *N*,*N*-diisopropylamino ethylamine; DiR: 1,1′-dioctadecyl-3,3,3’,3′-tetramethyl indotricarbocyanine iodide; DNA: deoxyribonucleic acid; DOX: doxorubicin; DPPC: dipalmitoyl phosphatidylcholine; DSPE: 1,2-distearoyl-*sn*-glycero-3-phosphoethanolamine; DSPE-PEG: 1,2-distearoyl-*sn*-glycero-3-phosphoethanolamine-poly(ethyleneglycol); EPR: enhanced permeability and retention; FA: folic acid; FDA: Food and Drug Administration; Fe_3_O_4_: magnetite nanoparticles; Fe_3_O_4_-iSWNTs: single wall carbon nanotubes doped with magnetite nanoparticles; Gal: Galactosyl; GCPQ: *N*-palmitoyl-*N*-monomethyl-*N*,*N*-dimethyl-*N*,*N*,*N*-trimethyl-6-O-glycolchitosan; GSH: glutathione; HA: hyaluronic acid/hyaluronan; Hesp: hesperetin; IC_50_: half-maximal inhibitory concentration; IL- 1β/-6/-12: interleukin- 1β/-6/-12; Lac: lactosyl; LCST: lower critical solution temperature; LF: lactoferrin; lncRNA: long noncoding RNA; MEA: 2-aminoethanethiol; MHT: magnetic hyperthermia; MnFe_2_O_4_ (s, c): MnFe_2_O_4_ nanoparticles with spherical and cubic shapes; MnFe_2_O_4_@Fe_3_O_4_: MnFe_2_O_4_ nanoparticles in the core with a shell of Fe_3_O_4_; MNPs: magnetic nanoparticles; MPMs: magnetic polymeric micelles; MRI: magnetic resonance imaging; MS: saturation magnetization; NA: nucleic acids; NIR: near infrared; NPX: naproxen; NSCs: neural stem cells; OAMAM: oligo (amidoamine) dendron; OAMAM-*b*-DEX: oligo (amidoamine) dendron-*block*-dextran; OA-R837: oleic acid-modified imiquimod; OCL-Bz-*b*-PEG: oligo(ɛ-caprolactone)-benzoyl-*block*-poly(ethylene glycol); Octyl-HTCC: Octyl-grafted-*N*-(2-hydroxy) propyl-3-trimethyl ammonium chitosan chloride; Octyl-g-PEG-HTCC: Octyl-grafted-poly(ethylene glycol)-*N*-(2-hydroxy) propyl-3-trimethyl ammonium chitosan chloride; OVA: amine-containing ovalbumin antigen; PA: palmitic acid; PAA: poly(acrylic acid); P(AAm-*co*-AN): poly(acrylamide-*co*-acrylonitrile); P(AAm-*co*-AN)-*g*-PEG: poly(acrylamide-co-acrylonitrile)-*grafted*-poly (ethylene glycol); PAE: poly(β-amino ester); PAE-b-PEG: poly(β-amino ester)-block-poly(ethylene glycol); PAE-PEG/DPPC: poly(β-amino ester)-*block*-poly(ethylene glycol) and dipalmitoyl phosphatidylcholine; PAELG: poly(2-azidoethyl-L-glutamate); PAMPs: pathogen-associated molecular pattern; PAsp: poly(L-aspartic acid); PAsp(DBA-*co*-DIP)-b-PEG: poly(N-(*N*,*N*-dibutalaminoethyl-*co*-*N*,*N*-diisopropylaminoethyl)aspartamide)-*block*-poly(ethylene glycol); PAsp(DMA): poly(aspartic acid)-dimethylethanediamine; PAsp(MEA-*co*-DIP)-*b*-PEG: poly(*N*-(2-aminoethanethiol-*co*-2-aminoethyldiisopropylamine)aspartamide)-*block*-poly(ethylene glycol); PBA: phenylboronic acid; PBLA: poly(β-benzyl L-aspartate); PCCL-*b*-PEG: poly(carboxyl-ε-caprolactone-*block*-poly(ethylene glycol)); PCL: poly(ε-caprolactone); PCL-*b*-HA: poly(ε-caprolactone)-*block*-hyaluronan; PCL-*b*-PAELG: poly(ε-caprolactone)- *block* -poly(2-azidoethyl-L-glutamate); PCL-*b*-PEG: poly(ε-caprolactone)-*block*-poly(ethylene glycol); PCL-*b*-PGA: polycaprolactone-*block*-poly(glutamic acid); PCys(SO_2_Et): poly(S-ethylsulfonyl-L-cysteine); PCys(SO_2_Et)-*b*-PSar: poly(S-ethylsulfonyl-L-cysteine)-*block*-polysarcosine; PDI: polydispersity index; PDLA-*b*-PEG/PLLA-*b*-PEG: blending of poly(ethylene glycol)-*block*-poly(D-lactide) and poly(ethylene glycol)-*block*-poly(L-lactide); PDT: photodynamic therapy; PEG: poly(ethylene glycol); PEG-PU-PCL-PU-PEG: multiblock polyurethane; PEI: poly(ethylenimine); PEO-PPO-PEO: poly(ethylene oxide)-*block*-poly(propylene oxide)-*block*-poly(ethylene oxide) (Pluronic F127); PGA: poly(glutamic acid); PHEMA: poly(2-hydroxymethyl) methacrylate; PHEMA-*b*-PEG: poly(2-hydroxymethyl) methacrylate-*block*-poly(ethylene glycol); PHEP: poly(2-methoxy-2-oxo-1,3,2-dioxaphospholane); PHEP-*b*-PEG: poly(2-hexoxy-2-oxo-1,3,2-dioxaphospholane)-*block*-poly(ethylene glycol); pI: isoelectric point; PLA: polylactic acid; PLA-*b*-PEG: poly(lactic acid)-*block*-poly(ethylene glycol); PLA-PEG/PLA-CHI-Spm: combination of PLA-PEG and PLA-Chitosan-Spermine; PLGA: poly(lactide-*co*-glycolic acid); PLGA-*b*-PEI-*b*-PEG: poly(lactic-*co*- glycolic acid)-*block*-poly(ethylenimine)-*block*-poly(ethylene glycol); PMs: polymer micelles; PNIPAM: poly(*N*-isopropylacrylamide);PNIPAM-PCL-PNIPAM:poly(*N*-isopropylacrylamide)-*block*-poly(caprolactone)-*block*-poly(*N*-isopropylacrylamide); P(NIPAAm-*co*-DMAAm-*co*-UA): poly(*N*-isopropylacrylamide-*co*-*N*,*N*- dimethylacrylamide-*co*-10-undecenoic acid); PPI: poly(phenyl isocyanide); PPI-*b*-TEGME: poly(phenyl isocyanide)-*block*-triethylene glycol monomethyl ether; PPO: poly(propylene oxide); PS: poly(styrene); PS-*b*-DNA: poly(styrene)-*block*-deoxyribonucleic acid; PS-*b*-PAA-*b*-PEG: poly(styrene-*block*-acrylic acid-*block*-ethylene oxide); PSar: polysarcosine; Pt(IV): platinum(IV); PTX: paclitaxel; P4VP: poly(4-vinylpyridine); P4VP-*b*-PEG: poly (4-vinylpyridine)-*block*-polyethylene glycol; PZLL: poly-(*N*-ε-carbobenzyloxy-L-lysine); PZLL-*g*-HA: poly-(*N*-ε-carbobenzyloxy-L-lysine)-*grafted*-hyaluronan; QCT: quercetin; *r*_2_: transverse relaxation coefficient; RNA: ribonucleic acid; SF: sorafenib; siRNA/ASO: small interfering RNA/antisense oligonucleotides; SPIONs: superparamagnetic iron oxide nanoparticles; Spm: spermine; *T*_2_: transverse relaxation time; TAM: tamoxifen; TEGME: triethylene glycol monomethyl ether; TERM: tissue engineering and regenerative medicine TLRs: Toll-Like Receptors; TNF-α: tumor necrosis factor-alpha; UA: 10-undecenoic acid; UCST: upper critical solution temperature; Y_1_R: Y_1_R receptor ligand.
